# Exploring the sequence fitness landscape of a bridge between protein folds

**DOI:** 10.1371/journal.pcbi.1008285

**Published:** 2020-10-13

**Authors:** Pengfei Tian, Robert B. Best

**Affiliations:** Laboratory of Chemical Physics, National Institute of Diabetes and Digestive and Kidney Diseases, National Institutes of Health, Bethesda, Maryland, U.S.A; Penn State College of Medicine, UNITED STATES

## Abstract

Most foldable protein sequences adopt only a single native fold. Recent protein design studies have, however, created protein sequences which fold into different structures apon changes of environment, or single point mutation, the best characterized example being the switch between the folds of the GA and GB binding domains of streptococcal protein G. To obtain further insight into the design of sequences which can switch folds, we have used a computational model for the fitness landscape of a single fold, built from the observed sequence variation of protein homologues. We have recently shown that such coevolutionary models can be used to design novel foldable sequences. By appropriately combining two of these models to describe the joint fitness landscape of GA and GB, we are able to describe the propensity of a given sequence for each of the two folds. We have successfully tested the combined model against the known series of designed GA/GB hybrids. Using Monte Carlo simulations on this landscape, we are able to identify pathways of mutations connecting the two folds. In the absence of a requirement for domain stability, the most frequent paths go via sequences in which neither domain is stably folded, reminiscent of the propensity for certain intrinsically disordered proteins to fold into different structures according to context. Even if the folded state is required to be stable, we find that there is nonetheless still a wide range of sequences which are close to the transition region and therefore likely fold switches, consistent with recent estimates that fold switching may be more widespread than had been thought.

## Author summary

While most proteins self-assemble (or “fold”) to a unique three-dimensional structure, a few have been identified that can fold into two distinct structures. These so-called “metamorphic” proteins that can switch folds have attracted a lot of recent interest, and it has been suggested that they may be much more widespread than currently appreciated. We have developed a computational model that captures the propensity of a given protein sequence to fold into either one of two specific structures (GA and GB), in order to investigate which sequences are able to fold to both GA and GB (“switch sequences”), versus just one of them. Our model predicts that there is a large number of switch sequences that could fold into both structures, but also that the most likely such sequences are those for which the folded structures have low stability, in agreement with available experimental data. This also suggests that intrinsically disordered proteins which can fold into different structures on binding may provide an evolutionary path in sequence space between protein folds.

## Introduction

There is an enormous variety of protein sequences found in nature, with around 170 million non-redundant sequences registered in the Refseq database [1] at the time of writing. A significant fraction of these, approximately 1/3 in eukaryotes [2, 3], are intrinsically disordered. The sequence diversity of the remainder, which fold to a specific structure, belies a simplicity in the structures to which they fold: most folded proteins can be classified into one or more independently folding units, or domains [4], and the number of domains which have a distinct structure, numbering in the thousands, is much more limited than the number of sequences that fold to these structures [5, 6]. Here, by distinct structure, we mean proteins which have the same overall fold, i.e. that the three dimensional arrangement of the backbone and secondary structure elements is similar. While the number of experimentally determined structures in the protein data bank continues to grow rapidly, the number of known folds is increasing only very slowly, suggesting that most existing naturally occurring folds are already known [6].

Recent advances in protein design have also shown it is possible to design completely novel folds, not observed in nature [7]. Therefore the number of folds sampled by evolution is smaller than the number possible. Indeed a molecular simulation study exploring possible protein architectures hinted that the number of possible folds may even be considerably larger than those currently known [8]. These results, as well as bioinformatics analysis [9], suggest that the emergence of new folds is a very rare event in protein evolution. How, then, do new folds arise? One possible route is via evolution of existing ones [10, 11]. In this scenario, there would be pathways in sequence space between the two folds, in which the intermediate sequences would have some propensity to fold into both structures. Such sequences are expected to be very rare, given that the fraction of possible random sequences which actually fold to a specific, stable backbone structure is already extremely tiny [12–18]. An initial suggestion that such sequences may be possible comes from the context dependence of secondary structure elements [19, 20] and since internal loops linking these elements are agnostic to secondary structure, they can also be shared between different topologies [21–23].

Remarkably, there are indeed several naturally occurring examples in which the same protein sequence can adopt two completely different stable folds apon changes in conditions [24], for example changes in pH (lymphotactin [25]), or binding to another molecule (KaiB [26]). It has also been possible to design proteins which can switch folds: a temperature-sensitive local switch of structure between helix and sheet was obtained in a designed version of arc-repressor [27, 28], and more recently sequences have been designed which make the dramatic switch between the all-*α* GA and *α*/*β* GB folds of streptococcal protein G apon single-point mutation, or addition of a binding partner [24, 29]. These so-called “metamorphic” proteins [30] have sparked interest for their biophysical properties, their potential roles as molecular switches, as well as their possible link to protein evolution. Bioinformatics analysis has suggested that such fold switches may be even more widespread than currently thought [31, 32].

**Fig 1:**
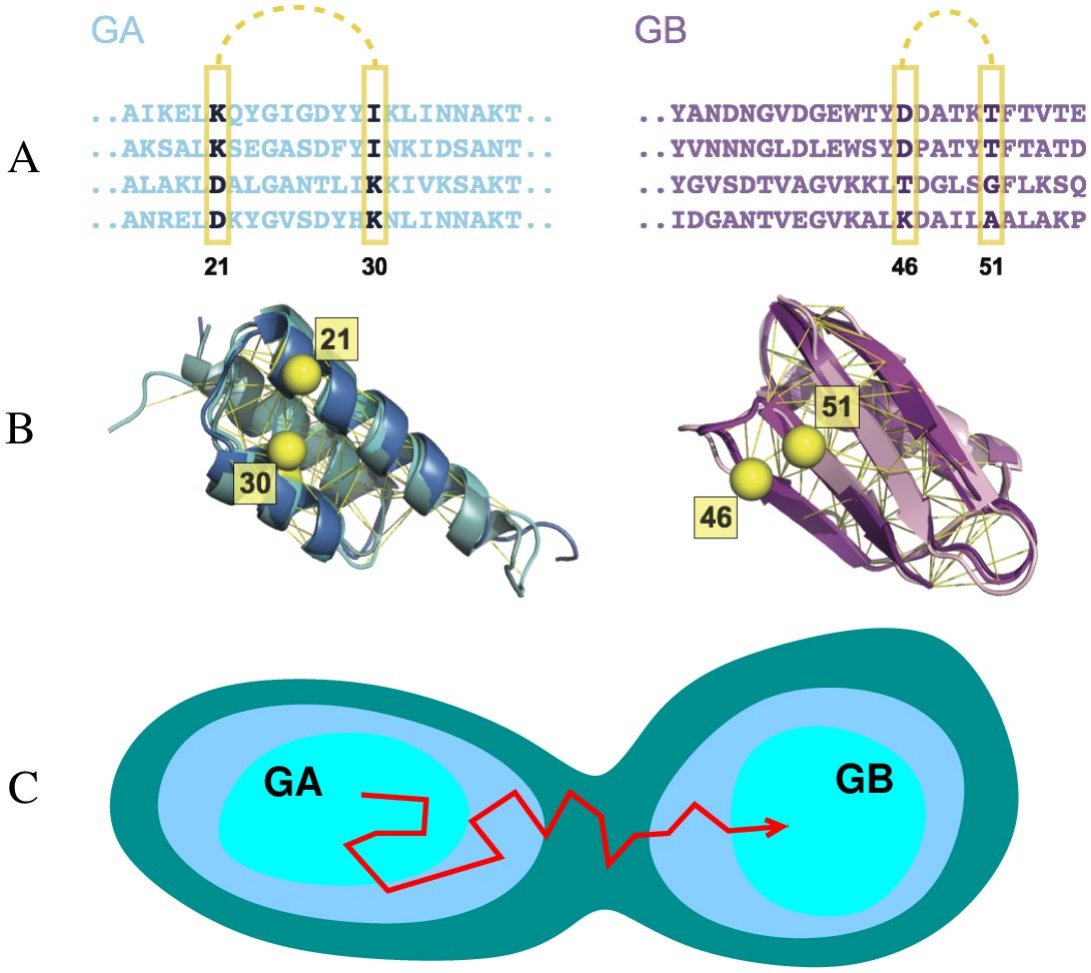
Sequence-based models for the GA and GB domains of streptococcal protein G. Many sequences (A) fold to each structure (B): e.g. structures of three naturally occurring sequences with the GA fold (pdb ID 2fs1, 1gjs and 2j5y) and three with the GB fold (pdb ID 1pga, 2lum and 1igd) are shown on the left and right respectively. Contacts between pairs of residues in the native structure (C*β* atoms of example pairs in yellow) impose mutual constraints on the types of residues which can occupy these positions in the sequence alignment. For instance, strong covariance is detected between the amino acids at residue 21 and 30 for GA sequences and between residues 46 and 51 for GB sequences. The C*β* atoms of these residues are illustrated in yellow sphere. The UniProtKB ID of these example sequences for GA are Q51918_FINMA, G5KGV3_9STRE, G5K7M6_9STRE and Q56192_STAXY. And the ones for GB are SPG1_STRSG, E4KPW8_9LACT, F9P4J6_STRCV and G5JZF8_9STRE. (C) Simple model for the emergence of new folds via evolutionary drift in sequence space between basins of attraction corresponding to the GA and GB domains.

The designed fold switch between the all-*α* GA and the *α*/*β* GB folds is the best experimentally characterized metamorphic protein pair (Fig 1). Via a systematic, and conservative, alteration of the sequence, Bryan, Orban and co-workers have demonstrated that it is possible to switch the structure of the GA domain (Fig 1 green) to the GB domain (Fig 1 purple) [33]. In some cases, a single point mutation is enough to switch from one structure to another, and some variants appear to be able to populate both structures, under different conditions [29]. The rich structure and stability data describing a mutational pathway between the GA and GB folds has inspired a number of theoretical studies of the fold switching phenomenon [34]. The models used in such studies are, by necessity, usually highly simplified: for example, a reduced three-letter protein model was used to study the sharp fold switch caused by a short mutational path [35]. 2-D lattice models can also be used as generic models to explore the general features of sequences that act like evolutionary bridges [36, 37]. The above models attempt to model both the changes in sequence space, as well as the actual folding of the chain in three dimensions. This requirement necessarily limits them to model systems (reduced alphabets, lattice models). In order to describe and predict protein sequences which act as a bridge between the specific GA and GB folds, a more detailed model is needed. One approach is to use all-atom physical force fields [38, 39], but these are very computationally expensive and still not fully predictive. By combining an all-atom physical force field with an additional energy term for native contacts it was possible to determine the free energy differences between fold switch mutants [40]. However, adjusting the relative weight between the native contacts energy and physical energy is not trivial, and the application is limited to a few mutants due to the computational cost involved. Some sequence-dependent models have been parametrized to fit the fold propensity of the mutations at the interface of the GA/GB fold, but the overall landscape of the bridge between two folds was not characterized [39, 41].

Our goal in this work was to develop a model for a sequence-space fitness landscape representing the joint fitness for the GA and GB folds, and to characterize pathways in sequence space between folds (Fig 1C). We use as input the observed sequence variation of protein homologues, which captures the covariation of amino acids at different sites; previously, we have demonstrated that it is possible to use such models to predict the effects of mutations for the proteins we have considered [14], as well as other those from previous studies [42–45]. We have even shown that it is possible to use such models to design novel sequences that fold stably into either a GA, GB or SH3 fold, representing the three basic classes of protein structure (all-*α*, *α*/*β*, all-*β* respectively) [46]. Here, we generalize such coevolutionary models to allow for transitions between the basins of attraction in sequence space corresponding to each fold. By using Monte Carlo simulations to sample transitions between these basins, we have described the characteristics of the mutational bridge between the GA and GB folds in sequence space. The rapid exploration of sequence space made possible with such a model allows us to investigate the effect that different requirements on the protein stabilities have on evolutionary dynamics [47, 48].

## Results

### Statistical model of GA and GB sequences

Maintaining the structure of the folded state is an important constraint on natural selection in protein evolution [42, 49, 50]. Therefore, proteins from the same family, which share the same fold, should contain common features in their sequences, both in the propensities of residues to be at certain positions, as well as the covariation between different sites which are in contact in the native state. The variation of the related sequences contained in a multiple sequence alignment (MSA) contains rich evolutionary information about structural and functional constraints (Fig 1).

In our work, we have built a model for the fitness of a given sequence to fold into a given structure, based on the covariation of sequences sampled in nature. The model for each protein family is parameterized using residue-residue coevolutionary information, which has previously been used to predict native contacts of protein structures [51–55], protein-protein interations [56–58] and RNA structures [59, 60]. Firstly, as shown in the MSA fragment in Fig 1, there is a propensity for certain residues to be found at a given position of the sequence. Secondly, there are correlations between the propensity at different sites, i.e. if one residue mutates, the proximal residues in the three dimensional structure will also likely mutate to maintain compatible physical and chemical interactions [61] (e.g. having Asp at position 21 and Lys at position 30 is favourable, but if position 21 is changed to Lys, it is unfavourable to have Lys at position 30). These propensities are approximated by the following Potts-like likelihood function *P*(*A*_1_, *A*_2_, ‥, *A*_*L*_), representing the likelihood of a given amino acid sequence *A*_1_, *A*_2_, ‥, *A*_*L*_, of length *L* for a particular protein fold,

$$\begin{array}{c} P\left(A_{1},A_{2},..,A_{L}\right)=\frac{1}{Z}\text{exp}\{\sum_{i<j}J_{ij}\left(A_{i},A_{j}\right)+\sum_{i}h_{i}\left(A_{i}\right)\}, \end{array}$$ (1)

In this function, the parameters *h*_*i*_ represent the single-site propensities for a given amino acid *A*_*i*_ at position *i*, while *J*_*ij*_ represents the propensity for amino acids *A*_*i*_ and *A*_*j*_ to be at positions *i* and *j*. These parameters are optimized to be consistent with the sequences observed in the MSA, using a pseudolikelihood optimization scheme [62]. From this probability, we associate an energy (“evolutionary Hamiltonian”) with a given sequence *x*, via *E*_EH_(*x*) = −ln *P*(*x*) (in units of *k*_B_*T*). We have built such a model for both protein families GA and GB. *E*_EH,GA_ and *E*_EH,GB_ are the two Hamiltonians inferred from the homologous sequences of GA and GB respectively using Eq 1; in earlier work, we showed that it was possible to design stably folded proteins using such evolutionary energy functions, for each of GA, GB and SH3 domains [46]. Others have shown that evolutionary energy functions can also be used for enzyme design [18]. Future testing on other domains will help to establish the generality of this approach. We first verified that Metropolis Monte Carlo simulations using the evolutionary energies *E*_GA_ or *E*_GB_ can recapitulate both the energy distribution of the sequences from the MSA of GA or GB (S1 Text Fig. A) as well as the amino acid composition frequencies (S1 Text Fig. B).

**Fig 2:**
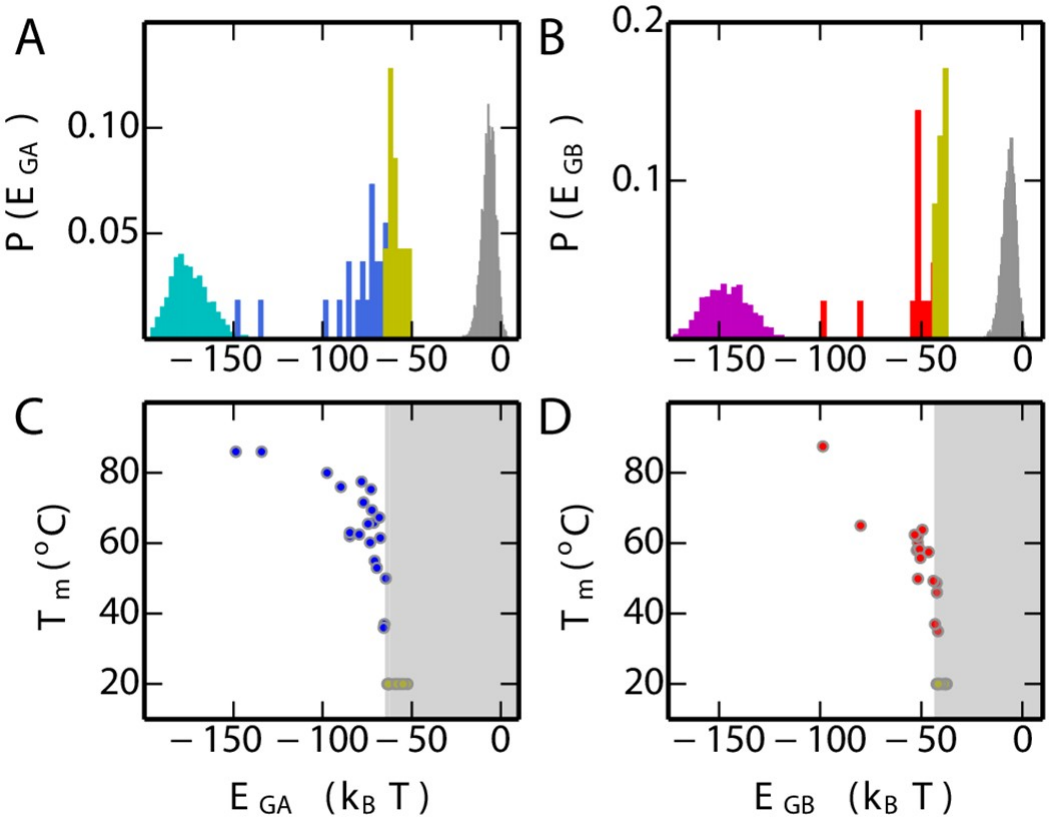
Properties of the single-fold models. (A) Distribution of *E*_GA_ for the GA homologs used to parameterize *E*_GA_ (cyan), synthetic sequences which are dominated by GA fold (blue) state in equilibrium, unstable synthetic sequences (yellow) and randomly generated sequences (grey). (B) Distribution of *E*_GB_ for the GB homologs used to parameterize *E*_GB_ (purple), synthetic sequences which are dominated by GB fold (red) state in equilibrium, unstable synthetic sequences (yellow) and random sequences (grey). (C) The correlation between the folding temperature (*T*_*m*_) and *E*_GA_ for synthetic sequences of GA. Stable mutants are blue symbols, unstable are yellow symbols with *T*_*m*_ set to 20°C for plotting purposes. (D) The correlation between *T*_*m*_ and *E*_GB_ for experimental mutants of GB (stable: red, unstable: yellow, *T*_*m*_ set to 20°C).

Some properties of the potentials are illustrated in Fig 2. As expected, the sequences used to build the model occupy the lowest energy region in each case (Fig 2A and 2B). The synthetic sequences designed by Bryan and co-workers (S1 Text, Table A) [29, 63–65] can be divided into those which are unstable, which have the highest energy with either *E*_GA_ or *E*_GB_, and those which fold to either GA or GB, which have energies intermediate between the respective training set and those that do not fold. We have also calculated the energies of sequences which we have generated by selecting at random from the residues which occur at each position in the sequence alignment, i.e. with no energy bias (grey histogram in Fig 2A and 2B). It is clear that the unstable designed sequences still have a significant propensity for the target fold, since their energies are much closer to the stable designed sequences than to random sequences. In Fig 2C and 2D, we compare the folding midpoint temperature *T*_*m*_ (data in S1 Text, Table B), a measure of folded state stability, and the statistical energy for each sequence. We observe a good correlation in each case (rank correlation coefficients of 0.86 and 0.92 for GA and GB respectively), with the unstable sequences also having the highest statistical energy. Such a correlation is expected if protein stability is an important consideration for natural selection, and has been observed also for other proteins [42–44]. In S1 Text Fig. C we show that a similar correlation exists with folding free energies, where those are available. Note that in addition to positive design, favoring a specific fold, coevolutionary models in principle should also capture negative design features, such as avoiding misfolding with adjacent domains [66–68]. To date, however, this aspect of these models has not been as well characterized as their ability to capture positive design features such as protein stability.

### A Combined fitness landscape for two protein folds

The models for GA and GB separately describe the fitness of sequences for each fold. In order to realize our goal of studying transitions between sequences which fold into GA and those which fold into GB, we require a single energy surface. A natural way to achieve this is to add the individual likelihood functions exp[−*E*_GA_] and exp[−*E*_GB_] or to use the more general combined energy function *E*_comb_ defined for sequence *x* as [69],

$$\begin{array}{c} e^{-\beta E_{\text{comb}}\left(x\right)}=\frac{1}{2}\left[e^{-\beta E_{\text{GA}}\left(x\right)}+e^{-\beta (E_{\text{GB}}\left(x\right)-\epsilon )}\right], \end{array}$$ (2)

where *β* is the inverse of a “mixing temperature” *T*_mix_ that determines the extent of mixing between the two potentials and is fixed here to 1.0. *ϵ* is an energy offset which sets the relative free energy of the two basins. Sequences from the GA MSA and GB MSA occupy the two minima of *E*_comb_, with the sequences near the barrier of the combined potential *E*_comb_ being putative “bridges” between the two folds.

**Fig 3:**
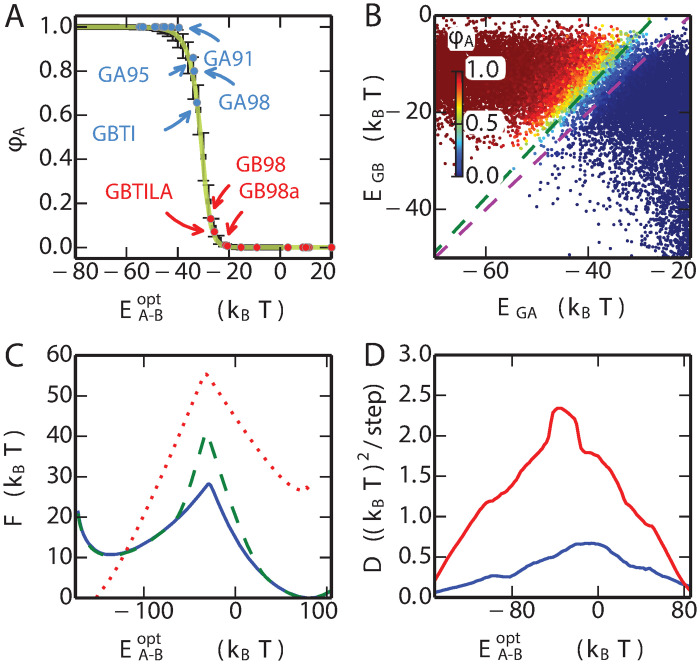
One-dimensional energy landscape capturing fold switch. (A) The committor for reaching the GA fold, *ϕ*_*A*_ is plotted for the experimentally characterized mutant sequences with blue (GA fold) and red (GB fold) symbols. The mean and standard deviation of *ϕ*_*A*_ for an equilibrium sample of sequences at given values of the optimized coordinate $E_{A-B}^{\text{opt}}$ are shown by black symbols and errorbars. The theoretical committor from a 1D diffusion model is shown in yellow. (B) The *ϕ*_*A*_ values (colours) are projected onto *E*_GA_ and *E*_GB_ for each sequence. Purple and blue broken lines are perpendicular to the original coordinate *E*_*A* − *B*_ = *E*_GA_ − *E*_GB_ and the optimized coordinate $E_{A-B}^{\text{opt}}=\lambda E_{\text{GA}}-E_{\text{GB}}$ respectively (λ = 1.13). (C) Free energy profile of the combined model for the natural mutations (blue), natural mutations with stability constraints (green) and the binary mutations (red). The free energy (in sequence space) was estimated using the weighted histogram analysis method, based on umbrella sampling on the coordinate $E_{A-B}^{\text{opt}}$. (D) The profile of position-dependent diffusion coefficients for the natural mutations (blue) and the binary mutations (red).

There is only one undetermined parameter in the combined energy function *E*_comb_, i.e. the offset energy *ϵ*. We find an appropriate value for *ϵ* using the committor function *ϕ*_A_(*x*) [70–72], defined as the probability that trial Monte Carlo simulations in sequence space (described in more detail below), initiated from sequence *x*, first reach the free energy minimum corresponding to the GA fold rather than GB: ideally sequences which are known to fold to GA should lie within the basin of attraction of GA in sequence space and have *ϕ*_A_ > 0.5, and those folding to GB would have *ϕ*_A_ < 0.5. An optimal *ϵ* = 23.0 is chosen for which the known propensity of a given sequence for the GA (versus GB) fold is correlated with the splitting probability *ϕ*_*A*_. With this choice, we find that *ϕ*_*A*_ is a good predictor of the favoured fold. Most of the designed sequences, such as GA30, GB30, GA77, GB77, GA88 and GB88, only ever populate one fold in experiment: Consistent with that, the *ϕ*_*A*_ estimated for these sequences is very close to 1.0 or 0.0. On the other hand, the mutants GA98, GB98, GB98-T25I and GB98-T25I/L20A all can adopt both GA and GB folds, either at equilibrium, or in the presence of binding partners. The GA fold is the most populated in the GA98 and GB98-T25I mutants, with a small population of the GB fold, ∼ 5% for GB98-T25I and ∼ 1% in GA98 [29]. For the GB98 and GB98-T25I/L20A mutants, the major population is the GB fold. The minor GA population in GB98 is larger than in GB98-T25I/L20A, although the exact populations have not been determined [29, 33]. The *ϕ*_*A*_ values of these four mutations in the Fig 3A, reproduce these observations, with *ϕ*_*A*_(GA98) > *ϕ*_*A*_(GB98-T25I) > 0.5 > *ϕ*_*A*_(GB98) > *ϕ*_*A*_(GB98-T25I/L20A). Note that alternative choices of *ϵ* will shift the position of the fold interface (i.e. *ϕ* = 0.5) while the relative ranking of *ϕ* over the different mutants is not changed (S1 Text Fig. D).

### Exploring fold switching in sequence space

Guided by the combined model *E*_comb_, we have explored the joint fitness landscape of the the two folds by the Monte Carlo simulation, in which a Metropolis criterion is used to accept or reject trial moves in sequence space. Such simulations correspond to a highly simplified model of protein evolution. We consider two different move sets in our simulations: “natural” and “binary” mutations. For natural mutations a new residue type is chosen with equal probability from those amino acids which are found at that position in the MSA of GA and GB. This restriction is made to avoid exploring regions of sequence space about which our statistical potential has no information and would therefore not be reliable. In the more conservative binary mutation scheme, the only allowed residues are those found in the reference GA and GB sequences (all of the sequences designed by Bryan et al. fall within this scheme [64]).

In order to characterize the fitness landscape, including regions with low population, we initially performed umbrella sampling using as reaction coordinate the energy gap *E*_A-B_(*x*) = *E*_GA_(*x*) − *E*_GB_(*x*), which has proved a useful coordinate in the context of previous problems involving mixed energy functions [73, 74]. This coordinate also separates quite well the sequences folding into GA vs GB (S1 Text Fig. E). In Fig 3B, we plot the sequences obtained from this sampling onto two variables, their statistical energies *E*_GA_ and *E*_GB_, with the point corresponding to each sequence coloured by its committor *ϕ*_A_. The committor *ϕ*_*A*_ is the probability that a Monte Carlo trajectory in sequence space, initiated from that sequence, will reach the free energy minimum associated with GA first, rather than reaching GB first. It has been proposed as an ideal reaction coordinate [71]. A corresponding committor *ϕ*_*B*_ can be defined for GB, from which it follows that *ϕ*_*B*_ = 1 − *ϕ*_*A*_. This plot shows a clear separation of the sequences falling into GA and GB basins of attraction (according to committor value), with the variation of committor approximately correlated with the energy gap. However, while the gap is certainly a reasonable choice, in this case it is not optimal for separating the two folds as it is clearly not exactly orthogonal to the dividing surface [75] (Fig 3B). An optimized version of the gap can be defined as $E_{\text{A-B}}^{\text{opt}}\left(x\right)=\lambda E_{\text{GA}}\left(x\right)-E_{\text{GB}}\left(x\right)$ in which the optimal value of λ is chosen to maximize the correlation of the coordinate with the committor value (illustrated in S1 Text Fig. F). In Fig 3C and 3D we plot the free energy and position-dependent diffusion coefficients obtained from our MC simulations, for this coordinate. As a separate check of the quality of the optimized reaction coordinate, we compare the average value of the committor computed assuming 1D dynamics with the actual average determined over the sequences at each value of the coordinate. The similarity of the two curves, in Fig 3A, demonstrates that $E_{\text{A-B}}^{\text{opt}}\left(x\right)$ is indeed a good reaction coordinate for describing the dynamics [76] (in contrast, the agreement is not good using the unoptimized energy gap, as shown in S1 Text Fig. G).

### What is the barrier to fold switching?

The barrier in the free energy on $E_{\text{A-B}}^{\text{opt}}\left(x\right)$ is a measure of the difficulty of finding a path in sequence space between the two folds. For both move sets, there is a substantial barrier, ∼ 15*k*_B_*T* for all natural mutations and ∼ 30*k*_B_*T* when allowing only binary mutations (Fig 3C). The higher barrier for binary mutations is anticipated due to the more restricted available sequences in that case. Although the dynamics we simulate is highly simplified as a model of protein evolution, the height of the free energy barrier, together with reasonable assumptions about the kinetic prefactor (based on replication error rates, population sizes and generation cycles), would suggest that this type of transition between folds is indeed a very rare event.

**Fig 4:**
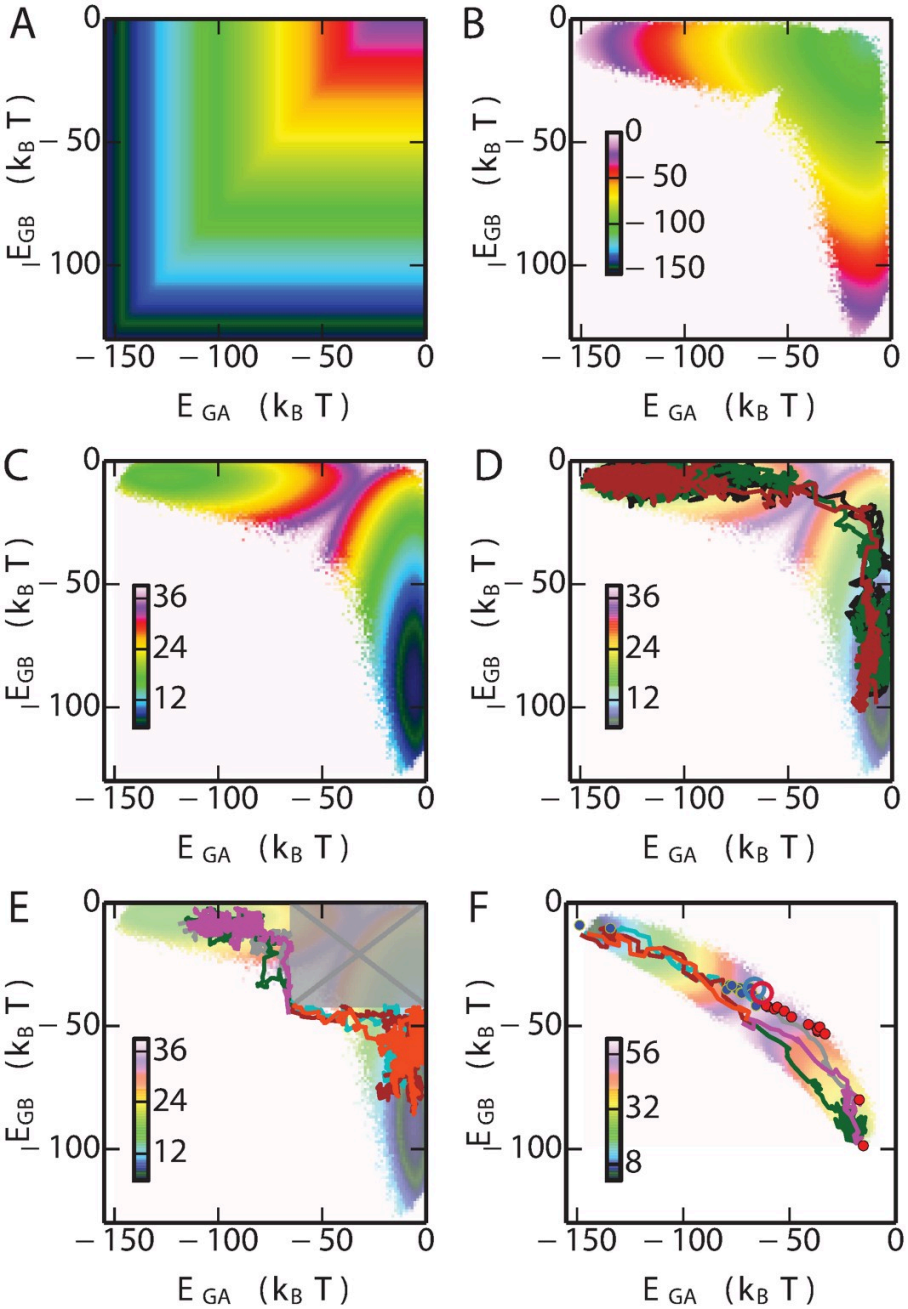
Fitness landscape. (A) Potential energy landscape of the combined model. (B) Contribution of entropy to free energy. (C) 2D free energy landscape of the fold switch for natural mutation simulations. (D) Example of three transition paths from GA basin to the GB basin. Examples of transition paths (E) with stability constraints (shaded and crossed box represents forbidden region where one or both folds is predicted to be unstable), and (F) using only “binary” mutations. The free energy surface in (F) is the one in which only binary mutations are allowed. All energies are in *k*_B_*T*.

To further investigate the origin of the free energy barrier between the GA and GB basins in sequence space, we calculated the 2-dimensional free energy landscape projected onto *E*_GA_ and *E*_GB_ (Fig 4C), based on umbrella sampling simulations in which all natural mutations were allowed. We see that the lowest free energy path from GA to GB does not follow a direct route, but rather an L-shaped path via a region where both *E*_GA_ and *E*_GB_ are large. In the context of our results on the correlation between protein stability and the statistical energies *E*_GA_ and *E*_GB_, the implication is that the most likely paths between folds go via unfolded, or unstable, states. We can obtain more insight into this by separating the free energy *F*(*E*_GA_, *E*_GB_) into its energetic *E*_comb_(*E*_GA_, *E*_GB_) and entropic *S*(*E*_GA_, *E*_GB_) = (*E*_comb_(*E*_GA_, *E*_GB_) − *F*(*E*_GA_, *E*_GB_))/*T* (Fig 4B). Although the minimum energy path would clearly favour a direct transition from GA to GB, the very large contribution from sequence entropy favours a path through disordered states [77, 78]. In retrospect, this result seems obvious, given the vast size of unconstrained sequence space, relative to the size of the regions in which folds such as GA and GB are stable.

### Transition paths between folds with and without stability as an evolutionary pressure

In addition to calculating free energy surfaces from umbrella sampling, we have also determined directly examples of likely transition paths between the GA and GB folds. Since the free energy barrier between the two folds is very high (Fig 3C), spontaneous transitions from one fold to another will rarely happen if using conventional sampling techniques. To obtain more statistics on the transitions, we used the transition path sampling technique (details in Methods), from which around 1000 transition paths on the fold bridge were obtained, a few of which are shown in Fig 4D (with the remainder in S1 Text Fig. H). Consistent with the free energy surfaces, all paths go via sequences which have high values of *E*_GA_ and *E*_GB_, suggesting that in the absence of a constraint on protein stability, the most likely transitions from one fold to another involve sequences with lowered propensity for either fold. However, the average energies of the sequences in the transition region in Fig 4D are still below zero, suggesting that some propensity for folding to GA and/or GB is retained even if the stability is low. We note that both experiments on GA/GB intermediates (see Fig 2) [29, 63, 64], and simulations of simplified models [35, 41], have also suggested that loss of stability is invariably obtained as one approaches the bridge between folds.

Because in the cell unfolded chains would ordinarily be rapidly degraded, and because many proteins must be folded in order to function, the above scenario of fold conversion might be considered unrealistic. To avoid sampling sequences which are predicted to be unstable, we have also run transition-path sampling simulations in which the values of *E*_GA_ and *E*_*GB*_ are constrained to be below the boundaries separating stable and unstable sequences, -64.6 and -41.7 *k*_B_*T* for GA and GB respectively (Fig 2C and 2D). The results of these runs, illustrated in Fig 4E, show that there are still many possible paths allowed even with this restriction, consistent with the experimental finding of multiple stable bridge sequences. Interestingly, when only binary mutations are allowed (Fig 4F), both the free energy surface and example transition paths suggest that the stability requirement is generally satisfied without having to be separately imposed. This follows from the much smaller sequence entropy contribution in this case; however, this restriction on sequence space also corresponds to a strong bias toward the target sequence. We note that the synthetic sequences on the fold bridge [29, 63, 64] (GA fold: blue, GB fold: red dot), also designed within the binary mutation space, fall within the bundle of transition paths sampled in this way (Fig 4F). In addition Elber and co-workers have computationally designed, using the binary sequence space, a pair of sequences S1 and S2 which differ at one residue but are predicted to adopt the GB and GA folds respectively [79]. According to our model, S1 and S2, are shown as red and blue hollow circles in Fig 4F, are very close to the fold interface. The *ϕ*_*A*_ of S2 and S1 are ∼ 1.0 and ∼ 0.87, respectively, suggesting that S2 has higher propensity to fold into GA topology than S1, consistent with the earlier prediction [79].

### Fold bridge sequences are likely to be intrinsically disordered

**Fig 5:**
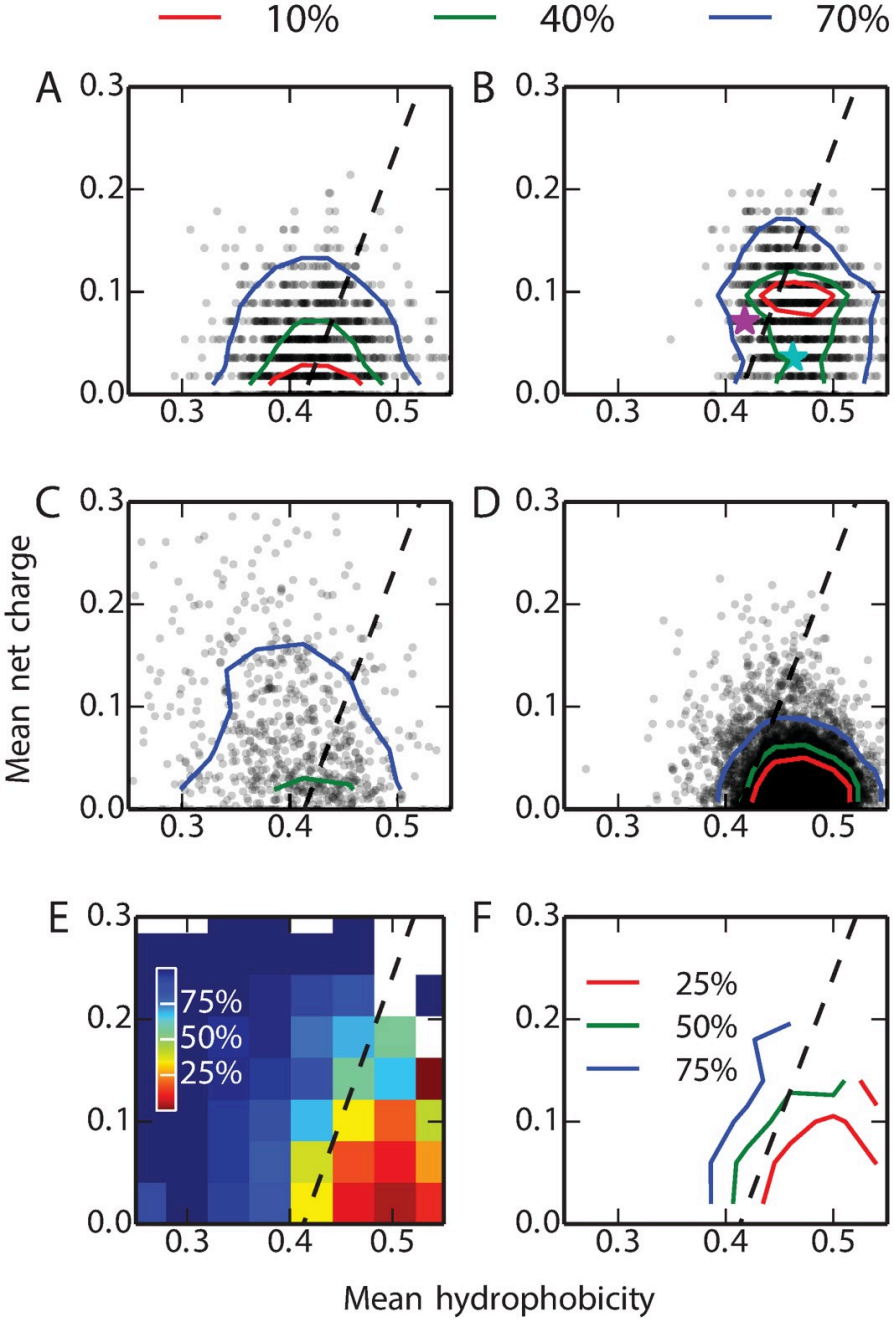
The Uversky plot divides proteins into folded globular and intrinsically disordered proteins based on their mean net change (*q*) and the mean hydrophobicity (*h*) [80]. In each plot, the dashed line represents the boundary between the two subsets described by Uversky [80]. We calculated the *q*, *h* of 10000 randomly selected transition sequences, defined as having *ϕ*_*A*_ within [0.49,0.51], from the simulations (A) without and (B) with stability constraints (one symbol for each sequence; probability density contours containing 10, 40 and 70% of the data are also shown). The *q*, *h* of 694 known IDPs from the DisProt database [81] and 7957 globular proteins from the Top8000 database [82] are shown in (C) and (D) respectively. Sequences of GA and GB wild-type are shown with cyan and purple stars, respectively, in (B). (E) and (F) are respectively heat map and contour map representations of the IDP propensity *P*(IDP|*q*, *h*). The legends (%) represent the probability of being an IDP *P*(IDP|*q*, *h*) for each (*q*, *h*) combination.

What are the physical properties of the switch sequences (those with a committor *ϕ*_*A*_ ≃ 0.5) obtained from our simulations? A simple classification into sequences which favour globular structures and those which are more likely to be intrinsically disordered can be made on the basis of the mean net charge, *q*, and mean hydrophobicity, *h*. We have mapped the switch sequences obtained from our model using natural mutations onto these coordinates: Fig 5A and 5B show, respectively, the results without and with a restraint on native state stability. On these plots, Uversky has determined that the line *q* = 2.785*h* − 1.151 [80] approximately separates IDP and globular sequences: by this criterion, 58% of the switch sequences without a restraint on native state stability fall into the IDP region, compared with only 26% when stability constraints are imposed. For reference, we have also calculated the *q* and *h* of experimentally well-characterized sequences from the IDP database DisProt [81] (Fig 5C), with minimum disordered length > 4.) and the globular protein database Top8000 [82] (excluding those where regions of the sequence were not resolved in the structure). We find that 73% of the IDPs from DisProt and 8% of the globular proteins from the Top8000 are on the side of IDP as shown in Fig 5C and 5D respectively.

It is clear from the reference data in Fig 5C and 5D that the dashed line does not strictly separate IDPs and folded proteins. We have also employed a continuous descriptor, namely the conditional probability of being an IDP sequence for given values of *q* and *h*, *P*(IDP|*q*, *h*) (computed as described in Methods): this shows that indeed *P*(IDP|*q*, *h*) ≃ 50% near the previously determined dashed line (Fig 5E and 5F). If we use a more conservative IDP descriptor, namely *P*(IDP|*q*, *h*) > 80%, we find 31% and 6% of the switch sequences within this region without and with stability constraints, respectively. For comparison, of the simulated sequences from the two free energy basins of GA and GB, 1% and 7%, respectively, were in the IDP region. We have computed average disorder propensities using the DisEMBL [83] tool, which also shows enhanced disorder propensity for the fold-switch sequences (S1 Text Fig. I).

Thus, by all measures considered, the switch sequences identified from our model without requiring the protein to be stable are enriched in sequences with a high propensity for disorder, a finding that mirrors earlier work using lattice models [78]. This presents an alternative possibility to the scenario in which the folded state is constrained to be stable for all of the sequences bridging the two folds: the concern regarding possible aggregation or misfolding could be relieved by instead populating sequences with intrinsically disordered properties, namely low hydrophobicity and high net charge. Although unstable, these sequences would still have some propensity to fold to either GA or GB, as evidenced from their energies *E*_GA_ and *E*_GB_ being much below those for random sequences. The fact that it is much easier to find “bridge sequences” which are disordered than those that are folded may help to explain a growing catalog of IDPs which are able to fold into different structures upon binding with different ligands or other proteins [84], while such a property is very rarely observed for proteins which are independently stable. We note that disordered proteins are believed to be more abundant in complex genomes, due to a decrease in effective population size [85, 86]. Whether evolution might take a similar route between folds is a matter of speculation, but an intriguing possibility nonetheless, considering the much greater probability of finding a path in this way. The possibility that disordered sequences may act as a bridge between protein folds is consistent with the role of loops as basic elements of protein structure [21–23].

### Key residues controlling fold switching

**Fig 6:**
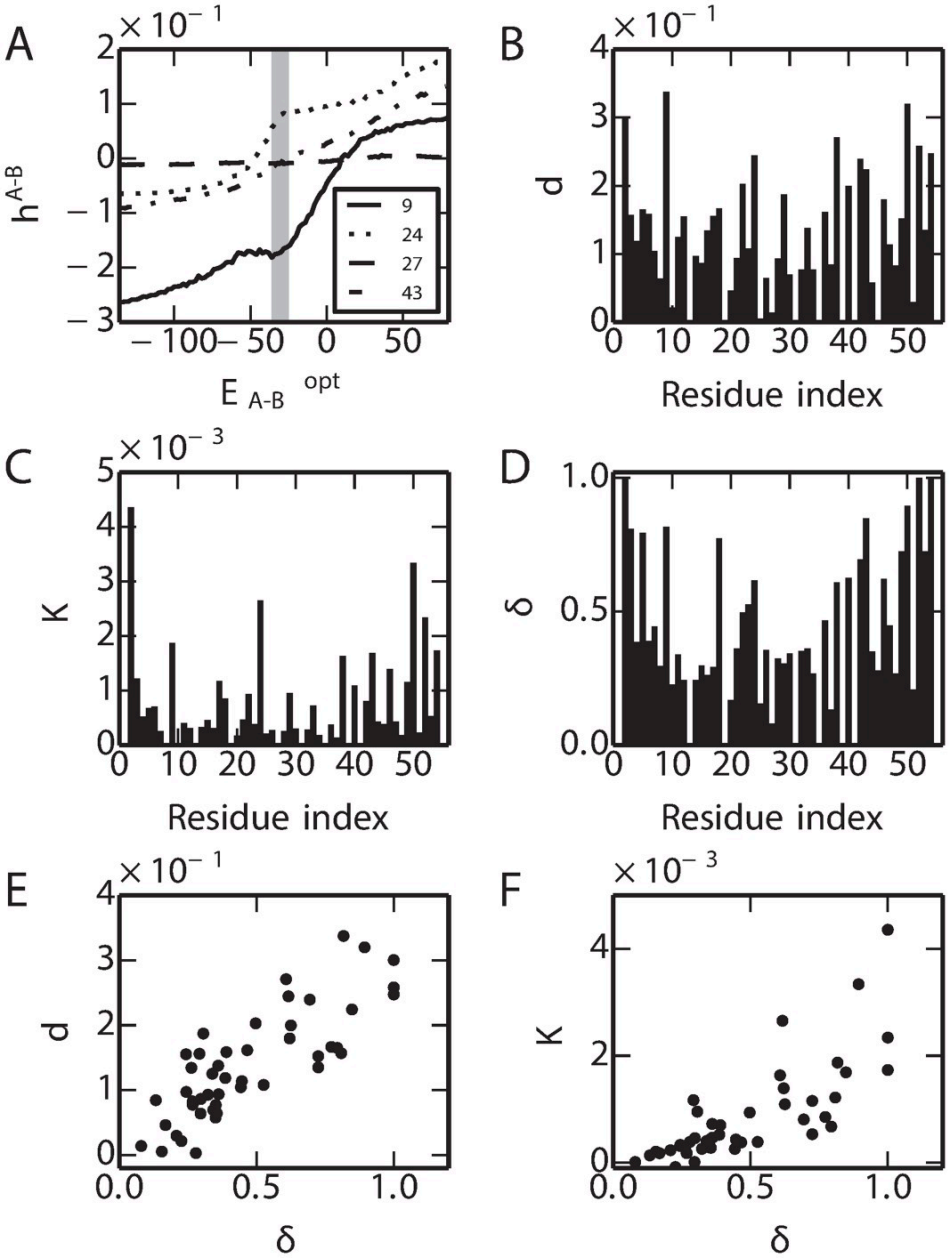
Single-site amino acid propensity changes in fold switching. (A) Examples of *h*^A-B^ for residues 9, 24, 27, 43. (B) Total change (*d*) of *h*^A-B^ from GA to GB. (C) Slope (K) at the transition region where it corresponds to *ϕ*_*A*_ ∈ [0.2,0.8]. The *δ* (D) and its correlation with *d* and *K* are show in (E) and (F).

An obvious question concerning the switch sequences is whether there are any key regions of the sequence which are more important in determining the switch from the GA to GB folds. Are there any common properties for the switch sequences? To identify the residues which play important roles for the fold switching, we analyzed the single site amino acid propensity during the fold switching when the stability constraints are imposed. The change of amino acid propensity from GA to GB sequence space at a given residue position can be indicated by $h_{i}^{\text{A-B}}=h_{i}^{\text{GA}}-h_{i}^{\text{GB}}$, where *i* is the residue index, $h_{i}^{\text{GA}}$ and $h_{i}^{\text{GB}}$ represent single-site propensities of GA and GB sequences respectively (Eq 1). The $h_{i}^{\text{A-B}}$ along the coordinate varies at different residues as shown in the Fig 6A. At each residue, the overall changes of $h_{i}^{\text{A-B}}$ (indicated by *d*) and the rate of change in the transition region (indicated by *K*) where $E_{\text{A-B}}^{\text{opt}}$ ∈ [-35.0, -33.0] (corresponding to *ϕ*_*A*_ ∈ [0.1, 0.9]), are shown in the Fig 6B and 6C respectively. At each residue position, to evaluate the similarity the probability distribution of the amino acid between the MSA of GA and GB, the Hellinger distance [87] (indicated by *δ*) is calculated as shown in Fig 6D. Interestingly, we found that there is strong correlation between *δ* to either *d* or *K*. It suggests that the residues which play important roles in the fold switching are the ones that have the most distinct amino acid compositions in the MSAs of GA and GB. We also analyzed $h_{i}^{\text{A-B}}$ when no stability constraints are imposed, leading to a similar conclusion (S1 Text Fig. J).

## Discussion

We have generated a simple sequence-based model which successfully captures the propensity of all experimentally characterized sequences to fold into either the GA or GB structures, as well as separating the stable from unstable sequences. We have previously validated sequences designed using such models experimentally [46]. By using an ansatz inspired from energy landscapes in configuration space, we have combined the sequence-based fitness landscapes of the two folds to create a joint fitness function that can describe the propensity for both folds. We have used Monte Carlo dynamics to sample this joint fitness landscape in order to identify sequences with similar propensity for both folds. Such sequences could be considered as transition states on evolutionary paths between the two folds. More concretely, such sequences should be those most likely to switch folds apon single point mutation or binding to a cognate ligand.

Our results suggest that the number of possible bridge sequences at the interface of two folds is potentially very large (Fig 4), even if the switch sequence is constrained to be stable (using the evolutionary Hamiltonian as a proxy for stability). Many of the bridge sequences generated from the simulation are predicted by the model to be of comparable or greater stability than the bridge sequences sampled in experiment [29, 63, 64]. The finding of multiple bridge sequences between folds may also be consistent with a recent analysis of the PDB suggesting that fold switching may be more common than previously thought [31].

Perhaps the most important conclusion from our study is that there are many more ways to find such fold-switch sequences which are unstable or have reduced stability. This is in qualitative accord with existing experimental and simulation studies [29, 35]. The reduction in stability may be expected to some extent based on the frustration between the sequence requirements of the two folds. Our study shows, however, that a second reason is the contribution from sequence entropy, which strongly favours a pathway via the more abundant low stability sequences. These low stability sequences tend to have properties usually associated with intrinsically disordered proteins (low hydrophobicity, higher charge content), raising the possibility that intrinsically disordered proteins may be able to function as bridges between protein folds in evolution [11, 88]. For example, there are several examples of IDPs that are known to fold to alternate structures when associating with different binding partners [84].

In future it will be interesting to apply this approach to design potential fold-switch sequences for this and other protein pairs which can be tested by experiment. In particular, it will be interesting to apply it to elucidating bistable coevolutionary models for naturally occurring fold-switching sequences [31], such as Lymphotactin [25] or KaiC [26]. More generally, such models could be used to assist in the prediction of previously unknown fold-switch proteins [32].

## Methods

### Multiple sequence alignments

The MSAs were generated with query sequences of GA (pdb code: 2FS1) [89] and GB (pdb code: 1PGA) [90] respectively, using the Jackhmmer method [91] (E-value cutoff: 10^−4^)) and the uniref90 database [92] (January, 2015). The MSAs contain 940 and 971 homologous sequences of GA and GB family respectively. The plmDCA method [62] was used to fit the likelihood function Eq 1 to the alignments. This method uses a weighting scheme for each sequence based on its similarity to the others to mitigate the effects of phylogenetic relationships on the results. The number of sequences included in the alignment is also an important factor, which we have investigated in our earlier work [14]. Either *E*_GA_ or *E*_GB_ can successfully distinguish the sequences from different families (S1 Text Fig. A). For instance, the sequences of GA family have much lower energy than sequences of GB family under function *E*_GA_, and vice versa.

### Monte Carlo sampling in the space of protein sequence

The Metropolis-Hastings Monte Carlo method is employed here for the sampling guided by the combined energy potential *E*_comb_. In each Monte Carlo iteration, the amino acid of one random residue is perturbed by a flip, from one type of amino acid to another. All allowed types of amino acid at that position are attempted with equal probability. This takes the system from one sequence *x*, with energy *E*_comb_(*x*), to a new sequence *x*′, with energy ${E_{\text{comb}}}^{\prime }\left(x^{\prime }\right)$. The move is accepted/rejected with acceptance probability

$$\begin{array}{c} P_{\text{acc}}=\text{min}\left[1,e^{-\beta ({E_{\text{comb}}}^{\prime }\left(x^{\prime }\right)-E_{\text{comb}}\left(x\right))}\right]. \end{array}$$ (3)

### First passage simulation and transition path sampling in sequence space

Transition states are critical to understand the transitionary bridge connecting the GA and GB families. In the first passage simulation, the MC simulations start from random sequences and stop when it reach the boundary of the reaction coordinate which correspondeds to either free energy minimum of the fold. However, due to the high free energy barrier, full transitions from one fold to another happen very rarely by conventional sampling within reasonable timescale. Therefore, statistics around the transition region is very hard to obtain. We use transition path sampling [93] to overcome this bottleneck by starting simulations from amino acid sequences on the top of the free energy barrier. Simulations are running until it hit the boundary of either free energy basin.

### Commmittors in sequence space

We have borrowed the concept of the committor from conventional statistical mechanics in configuration space [70, 71]. The committor for GA, *ϕ*_*A*_ is the probability that a trial Monte Carlo simulation in sequence space ends in the basin of attraction associated with GA, rather than that associated with GB. Consequently, the committor for GB, *ϕ*_*B*_, is related by *ϕ*_*B*_ = 1 − *ϕ*_*A*_. We estimate the committor for a given sequence by running 1000 Monte Carlo trials starting from that sequence and terminating when $E_{\text{A-B}}^{\text{opt}}<-110.0$ (GA basin reached) or when $E_{\text{A-B}}^{\text{opt}}>80.0$ (GB basin reached), and computing the proportion ending in the basin of interest.

### IDP propensity prediction

Given the mean net charge, *q*, and mean hydrophobicity, *h*, the probability of a sequence of being an IDP can be estimated from

$$\begin{array}{c} P\left(\text{IDP}|q,h\right)=\frac{P(q,h|\text{IDP})P(\text{IDP})}{P(q,h|\text{glob})P(\text{glob})+P(q,h|\text{IDP})P(\text{IDP})} \end{array}$$ (4)

where *P*(IDP) and *P*(glob) are the estimated probabilities of IDP and globular proteins in nature, which are set to 30% and 70% respectively [3]. *P*(*q*, *h*|glob) represents the joint distribution of *q* and *h* in globular proteins and *P*(*q*, *h*|IDP) the distribution for IDPs. Here, *P*(*q*, *h*|IDP) is obtained from 694 IDP sequences from the DisProt database [81] (Fig 5C) and the *P*(*q*, *h*|glob) is obtained from the 7957 Top8000 database of globular proteins [82] (Fig 5D).

## Supporting information

S1 Text Supporting text, tables and figures. Procedure for verifying likelihood model via MC simulations (Text A). Wild type and designed amino acid sequences (Table A). Summary of stability and melting temperature of wild-type and designed sequences from previous experiments (Table B). Distribution of energies from Monte Carlo simulations with evolutionary Hamiltonian (Fig. A). Frequencies of amino acids at each position from Monte Carlo simulations with evolutionary Hamiltonian (Fig. B). Correlation between evolutionary Hamiltonian and thermodynamic stability (Fig. C). Dependence of committor *ϕ*_*A*_ on *E*_comb_ (Fig. D). *E*_A-B_ of the designed sequences on the GA/GB fold interface (Fig. E). Determining optimal reaction coordinate from sequences with *ϕ*_*A*_ ≈ 1/2 (Fig. F). Quality of reaction coordinate assessed by comparison of true and calculated *ϕ*_*A*_ (Fig. G). Transition paths plotted on 2D fitness landscapes (Fig. H). Predictions from DisEMBL predictor (Fig. I). Residues controlling fold switching (Fig. J).
(PDF)

## References

[1] Pruitt K, Brown G, Tatusova T, Maglott D. The NCBI Handbook. Bethesda, MD: National Center for Biotechnology Information; 2012.

[2] Gsponer J, Futschik ME, Teichmann SA, Babu MM. Tight regulation of unstructured proteins: from transcript synthesis to protein degradation. Science. 2008;322:1365–1368. 10.1126/science.1163581 19039133 PMC2803065

[3] van der Lee R, Buljan M, Lang B, Weatheritt RJ, Daughdrill GW, Dunker AK, et al Classification of intrinsically disordered regions and proteins. Chem Rev. 2014;114:6589–6631. 10.1021/cr400525m 24773235 PMC4095912

[4] Vogel C, Bashton M, Kerrison ND, Chothia C, Teichmann SA. Structure, function and evolution of multidomain proteins. Curr Opin Struct Biol. 2004;14:208–216. 10.1016/j.sbi.2004.03.011 15093836

[5] Chothia C. One thousand families for the molecular biologist. Nature. 1992;357:543–544. 10.1038/357543a0 1608464

[6] Grant A, Lee D, Orengo C. Progress towards mapping the universe of protein folds. Genome Biol. 2004;5(5):107 10.1186/gb-2004-5-5-107 15128436 PMC416458

[7] Huang PS, Boyken SE, Baker D. The coming of age of de novo protein design. Nature. 2016;537:320–327. 10.1038/nature19946 27629638

[8] Cossio P, Trovato A, Pietrucci F, Seno F, Maritan A, Laio A. Exploring the universe of protein structures beyond the protein data bank. PLoS Comput Biol. 2010;6:e1000957 10.1371/journal.pcbi.1000957 21079678 PMC2973819

[9] Bukhari SA, Caetano-Annollés G. Origin and evolution of protein fold designs inferred from phylogenomic analysis of CATH domain structures in proteomes. PLoS Comput Biol. 2013;9:e1003009 10.1371/journal.pcbi.1003009 23555236 PMC3610613

[10] Davidson AR. A folding space odyssey. Proc Natl Acad Sci U S A. 2008;105(8):2759–2760. 10.1073/pnas.0800030105 18287054 PMC2268531

[11] Best RB. Bootstrapping new protein folds. Biophys J. 2014;107(5):1040–1041. 10.1016/j.bpj.2014.07.021 25185539 PMC4156660

[12] Koehl P, Levitt M. Protein topology and stability define the space of allowed sequences. Proceedings of the National Academy of Sciences. 2002;99(3):1280–1285. 10.1073/pnas.032405199 PMC122181 11805293

[13] Barton JP, Chakraborty AK, Cocco S, Jacquin H, Monasson R. On the entropy of protein families. J Stat Phys. 2016;162:1267–1293. 10.1007/s10955-015-1441-4

[14] Tian P, Best RB. How many protein sequences fold to a given structure? A co-evolutionary analysis. Biophys J. 2017;113:1719–1730. 10.1016/j.bpj.2017.08.039 29045866 PMC5647607

[15] Marchi J, Galpern EA, Espada R, Ferreiro DU, Walczak AM, Mora T. Size and structure of the sequence space of repeat proteins. PLoS Comput Biol. 2019;15:e1007282 10.1371/journal.pcbi.1007282 31415557 PMC6733475

[16] Facco E, Pagnani A, Russo ET, Laio A. The intrinsic dimension of protein sequence evolution. PLoS Comput Biol. 2019;15:e1006767 10.1371/journal.pcbi.1006767 30958823 PMC6472826

[17] Baker D. What has de novo protein design taught us about protein folding and biophysics. Protein Sci. 2019;28:678–683. 10.1002/pro.3588 30746840 PMC6423711

[18] Russ WP, Figliuzzi M, Stocker C, Barrat-Charlaix P, Socolich M, Hilvert PKD, et al An evolution-based model for designing chorismate mutase. Science. 2020;369(6502):440–445. 32703877 10.1126/science.aba3304

[19] Minor DL, Kim PS. Context-dependent secondary structure formation of a designed protein sequence. Nature. 1996;380:730–734. 10.1038/380730a0 8614471

[20] Cregut D, Civera C, Macias M, Wallon G, Serrano L. A tale of two secondary structure elements: when a *β*-hairpin becomes and *α*-helix. J Mol Biol. 1999;292:389–401. 10.1006/jmbi.1999.2966 10493883

[21] Berezovsky IN, Grosberg AY, Trifonov EN. Closed loops of nearly standard size: common basic element of protein structure. FEBS Lett. 2000;466:283–286. 10.1016/S0014-5793(00)01091-7 10682844

[22] Berezovsky IN, Guarnera E, Zheng Z. Basic units of protein structure, folding and function. Prog Biophys Mol Biol. 2017;128:85–99. 10.1016/j.pbiomolbio.2016.09.009 27697476

[23] Berezovsky IN. Towards descriptor of elementary functions for protein design. Curr Opin Struct Biol. 2019;58:159–165. 10.1016/j.sbi.2019.06.010 31352188

[24] Bryan PN, Orban J. Proteins that switch folds. Curr Opin Struct Biol. 2010;20(4):482–488. 10.1016/j.sbi.2010.06.002 20591649 PMC2928869

[25] Tuinstra RL, Peterson FC, Kutlesa S, Elgin ES, Kron MA, Volkman BF. Interconversion between two unrelated protein folds in the lymphotactin native state. Proc Natl Acad Sci U S A. 2008;105(13):5057–5062. 10.1073/pnas.0709518105 18364395 PMC2278211

[26] Chang YG, Cohen SE, Phong C, Myers WK, Kim YI, Tseng R, et al A protein fold switch joins the circadian oscillator to clock output in cyanobacteria. Science. 2015;349(6245):324–328. 10.1126/science.1260031 26113641 PMC4506712

[27] Cordes MHJ, Walsh NP, McKnight CJ, Sauer RT. Evolution of a protein fold in vitro. Science. 1999;284:325–327. 10.1126/science.284.5412.325 10195898

[28] Cordes MHJ, Burton RE, Walsh NP, McKnight CJ, Sauer RT. An evolutionary bridge to a new protein fold. Nat Struct Biol. 2000;7(12):1129–1132. 10.1038/81985 11101895

[29] He Y, Chen Y, Alexander PA, Bryan PN, Orban J. Mutational tipping points for switching protein folds and functions. Structure. 2012;20(2):283–291. 10.1016/j.str.2011.11.018 22325777 PMC3278708

[30] Murzin AG. Metamorphic proteins. Science. 2008;320(5884):1725–1726. 10.1126/science.1158868 18583598

[31] Porter LL, Looger LL. Extant fold-switching proteins are widespread. Proc Natl Acad Sci U S A. 2018;115:5968–5973. 10.1073/pnas.1800168115 29784778 PMC6003340

[32] Mishra S, Looger L, Porter LL. Inaccurate secondary structure predictions often indicate fold switching. Protein Sci. 2019;28:1487–1493. 10.1002/pro.3664 31148305 PMC6635839

[33] Bryan PN, Orban J. Implications of protein fold switching. Curr Opin Struct Biol. 2013;23(2):314 10.1016/j.sbi.2013.03.001 23518177

[34] Sikosek T, Chan HS. Biophysics of protein evolution and evolutionary protein biophysics. J Roy Soc Interface. 2014;11(100):20140419 10.1098/rsif.2014.0419 25165599 PMC4191086

[35] Holzgräfe C, Wallin S. Smooth functional transition along a mutational pathway with an abrupt protein fold switch. Biophys J. 2014;107(5):1217–1225. 10.1016/j.bpj.2014.07.020 25185557 PMC4156676

[36] Chan HS, Kaya H, Shimizu S. Computational methods for protein folding: scaling a hierarchy of complexities. Curr Topics Comput Mol Biol. 2002;p. 403–447.

[37] Sikosek T, Chan HS, Bornberg-Bauer E. Escape from Adaptive Conflict follows from weak functional trade-offs and mutational robustness. Proc Natl Acad Sci U S A. 2012;109(37):14888–14893. 10.1073/pnas.1115620109 22927372 PMC3443171

[38] Allison JR, Bergeler M, Hansen N, van Gunsteren WF. Current computer modeling cannot explain why two highly similar sequences fold into different structures. Biochemistry. 2011;50(50):10965–10973. 10.1021/bi2015663 22082195

[39] Chen SH, Elber R. The energy landscape of a protein switch. Phys Chem Chem Phys. 2014;16(14):6407–6421. 10.1039/c3cp55209h 24473276

[40] Sikosek T, Krobath H, Chan HS. Theoretical Insights into the Biophysics of Protein Bi-stability and Evolutionary Switches. PLoS Comput Biol. 2016;12(6):e1004960 10.1371/journal.pcbi.1004960 27253392 PMC4890782

[41] Sikosek T, Bornberg-Bauer E, Chan HS. Evolutionary dynamics on protein bi-stability landscapes can potentially resolve adaptive conflicts. PLoS Comput Biol. 2012;8(9):e1002659 10.1371/journal.pcbi.1002659 23028272 PMC3441461

[42] Morcos F, Schafer NP, Cheng RR, Onuchic JN, Wolynes PG. Coevolutionary information, protein folding landscapes, and the thermodynamics of natural selection. Proc Natl Acad Sci U S A. 2014;111(34):12408–12413. 10.1073/pnas.1413575111 25114242 PMC4151759

[43] Hopf TA, Ingraham JB, Poelwijk FJ, Schärfe CPI, Springer M, Sander C, et al Quantification of the effect of mutations using a global probability model of natural sequence variation. Nature Biotech. 2017;35:128–135. 10.1038/nbt.3769 PMC5383098 28092658

[44] Figliuzzi M, Jacquier H, Schug A, Tenaillon O, Weigt M. Coevolutionary landscape inference and the context-dependence of mutations in beta-lactamase TEM-1. Mol Biol Evolution. 2016;33(1):268–280. 10.1093/molbev/msv211 PMC4693977 26446903

[45] Cheng RR, Nordesjö O, Hayes RL, Levine H, Flores SC, Onuchic JN, et al Connecting the sequence-space of bacterial signaling proteins to phenotypes using coevolutionary landscapes. Molecular biology and evolution. 2016;33(12):3054–3064. 10.1093/molbev/msw188 27604223 PMC5100047

[46] Tian P, Louis JM, Baber JL, Aniana A, Best RB. Coevolutionary fitness landscapes for sequence design. Angew Chem Intl Ed. 2018;130:5776–5780. 10.1002/ange.201803004 PMC6147258 29512300

[47] Shakhnovich EI. Protein design: a perspective from simple tractable models. Folding and Design. 1998;3(3):R45–R58. 10.1016/S1359-0278(98)00021-2 9669878

[48] Manhart M, Morozov AV. Proc Natl Acad Sci U S A. 2014;112(6):1797–1802. 10.1073/pnas.1415895112 PMC4330747 25624494

[49] Li H, Helling R, Tang C, Wingreen N. Emergence of preferred structures in a simple model of protein folding. Science. 1996;273(5275):666–669. 10.1126/science.273.5275.666 8662562

[50] Zeldovich KB, Chen P, Shakhnovich EI. Protein stability imposes limits on organism complexity and speed of molecular evolution. Proceedings of the National Academy of Sciences. 2007;104(41):16152–16157. 10.1073/pnas.0705366104 PMC2042177 17913881

[51] Marks DS, Colwell LJ, Sheridan R, Hopf TA, Pagnani A, Zecchina R, et al Protein 3D structure computed from evolutionary sequence variation. PloS One. 2011;6(12):e28766 10.1371/journal.pone.0028766 22163331 PMC3233603

[52] Morcos F, Pagnani A, Lunt B, Bertolino A, Marks DS, Sander C, et al Direct-coupling analysis of residue coevolution captures native contacts across many protein families. Proceedings of the National Academy of Sciences. 2011;108(49):E1293–E1301. 10.1073/pnas.1111471108 PMC3241805 22106262

[53] Kamisetty H, Ovchinnikov S, Baker D. Assessing the utility of coevolution-based residue–residue contact predictions in a sequence-and structure-rich era. Proc Natl Acad Sci U S A. 2013;110(39):15674–15679. 10.1073/pnas.1314045110 24009338 PMC3785744

[54] Tian P, Boomsma W, Wang Y, Otzen DE, Jensen MH, Lindorff-Larsen K. Structure of a functional amyloid protein subunit computed using sequence variation. J Am Chem Soc. 2015;137(1):22–25. 10.1021/ja5093634 25415595

[55] Dauparas J, Wang H, Swartz A, Koo P, Nitzan M, Ovchinnikov S. Unified framework for modeling multivariate distributions in biological sequences. arXiv preprint arXiv:190602598. 2019.

[56] Schug A, Weigt M, Onuchic JN, Hwa T, Szurmant H. High-resolution protein complexes from integrating genomic information with molecular simulation. Proc Natl Acad Sci U S A. 2009;106(52):22124–22129. 10.1073/pnas.0912100106 20018738 PMC2799721

[57] Weigt M, White RA, Szurmant H, Hoch JA, Hwa T. Identification of direct residue contacts in protein–protein interaction by message passing. Proc Natl Acad Sci U S A. 2009;106(1):67–72. 10.1073/pnas.0805923106 19116270 PMC2629192

[58] Ovchinnikov S, Kamisetty H, Baker D. Robust and accurate prediction of residue–residue interactions across protein interfaces using evolutionary information. Elife. 2014;3:e02030 10.7554/eLife.02030 24842992 PMC4034769

[59] De Leonardis E, Lutz B, Ratz S, Simona C, Monasson R, Weigt M, et al RNA Secondary and Tertiary Structure Prediction by Tracing Nucleotide Co-Evolution with Direct Coupling Analysis. Biophys J. 2016;3(110):364a 10.1016/j.bpj.2015.11.1960

[60] Weinreb C, Riesselman AJ, Ingraham JB, Gross T, Sander C, Marks DS. 3D RNA and Functional Interactions from Evolutionary Couplings. Cell. 2016;165(4):963–975. 10.1016/j.cell.2016.03.030 27087444 PMC5024353

[61] Neher E. How frequent are correlated changes in families of protein sequences? Proc Natl Acad Sci U S A. 1994;91(1):98–102. 10.1073/pnas.91.1.98 8278414 PMC42893

[62] Ekeberg M, Lövkvist C, Lan Y, Weigt M, Aurell E. Improved contact prediction in proteins: using pseudolikelihoods to infer Potts models. Phys Rev E. 2013;87(1):012707 10.1103/PhysRevE.87.012707 23410359

[63] Alexander PA, He Y, Chen Y, Orban J, Bryan PN. The design and characterization of two proteins with 88% sequence identity but different structure and function. Proc Natl Acad Sci U S A. 2007;104(29):11963–11968. 10.1073/pnas.0700922104 17609385 PMC1906725

[64] Alexander PA, He Y, Chen Y, Orban J, Bryan PN. A minimal sequence code for switching protein structure and function. Proc Natl Acad Sci U S A. 2009;106(50):21149–21154. 10.1073/pnas.0906408106 19923431 PMC2779201

[65] Porter LL, He Y, Chen Y, Orban J, Bryan PN. Subdomain interactions foster the design of two protein pairs with 80% sequence identity but different folds. Biophys J. 2015;108(1):154–162. 10.1016/j.bpj.2014.10.073 25564862 PMC4286614

[66] Borgia MB, Borgia A, Best RB, Steward A, Nettels D, Wunderlich B, et al Single-molecule fluorescence reveals sequence-specific misfolding in multidomain proteins. Nature. 2011;474:662–665. 10.1038/nature10099 21623368 PMC3160465

[67] Tian P, Best RB. Structural determinants of misfolding in multidomain proteins. PLOS Comput Biol. 2016;12:e1004933 10.1371/journal.pcbi.1004933 27163669 PMC4862688

[68] Lafita A, Tian P, Best RB, Bateman A. TADOSS: computational estimation of tandem domain swap stability. Bioinformatics. 2019;35(14):2507–2508. 10.1093/bioinformatics/bty974 30500878 PMC6612889

[69] Best RB, Chen YG, Hummer G. Slow protein conformational dynamics from multiple experimental structures: the helix/sheet transition of arc repressor. Structure. 2005;13(12):1755–1763. 10.1016/j.str.2005.08.009 16338404

[70] Onsager L. Initial recombination of ions. Phys Rev. 1938;54:554–557. 10.1103/PhysRev.54.554

[71] Du R, Pande VS, Grosberg AY, Tanaka T, Shakhnovich ES. On the transition coordinate for protein folding. J Chem Phys. 1998;108(1):334–350. 10.1063/1.475393

[72] Geissler PL, Dellago C, Chandler D. Kinetic pathways of ion pair dissociation in water. J Phys Chem B. 1999;103:3706–3710. 10.1021/jp984837g

[73] Warshel A. Dynamics of reactions in polar solvents. Semiclassical trajectory studies of electron-transfer and proton-transfer reactions. J Phys Chem. 1982;86(12):2218–2224. 10.1021/j100209a016

[74] Chen YG, Hummer G. Slow conformational dynamics and unfolding of the calmodulin C-terminal domain. J Am Chem Soc. 2007;129:2414–2415. 10.1021/ja067791a 17290995

[75] Berezhkovskii A, Szabo A. One-dimensional reaction coordinates for diffusive activated rate processes in many dimensions. J Chem Phys. 2005;122:014503 10.1063/1.1818091 15638670

[76] Chodera JD, Pande VS. Splitting probabilities as a test of reaction coordinate choice in single-molecule experiments. Phys Rev Lett. 2011;107:098102 10.1103/PhysRevLett.107.098102 21929272 PMC4731355

[77] Zeldovich KB, Chen P, Shakhnovich BE, Shakhnovich EI. A first-principles model of early evolution: emergence of gene families, species, and preferred protein folds. PLoS Comput Biol. 2007;3:e139 10.1371/journal.pcbi.0030139 17630830 PMC1914367

[78] Gilson AI, Marshall-Christensen A, Choi JM, Shakhnovich EI. The role of evolutionary selection in the dynamics of protein structure evolution. Biophys J. 2017;112:1350–1365. 10.1016/j.bpj.2017.02.029 28402878 PMC5390048

[79] Chen SH, Meller J, Elber R. Comprehensive analysis of sequences of a protein switch. Protein Sci. 2016;25(1):135–146. 10.1002/pro.2723 26073558 PMC4815306

[80] Uversky VN. Natively unfolded proteins: a point where biology waits for physics. Protein Sci. 2002;11(4):739–756. 10.1110/ps.4210102 11910019 PMC2373528

[81] Sickmeier M, Hamilton JA, LeGall T, Vacic V, Cortese MS, Tantos A, et al DisProt: the database of disordered proteins. Nucleic Acids Res. 2007;35(suppl 1):D786–D793. 10.1093/nar/gkl893 17145717 PMC1751543

[82] Chen VB, Arendall WB, Headd JJ, Keedy DA, Immormino RM, Kapral GJ, et al MolProbity: all-atom structure validation for macromolecular crystallography. Acta Crystallographica Section D: Biological Crystallography. 2010;66(1):12–21. 10.1107/S0907444909042073 20057044 PMC2803126

[83] Linding R, Jensen LJ, Diella F, Bork P, Gibson TJ, Russel RB. Protein disorder prediction: implications for structural proteomics. Structure. 2003;11:1453–1459. 10.1016/j.str.2003.10.002 14604535

[84] Wright PE, Dyson HJ. Linking folding and binding. Curr Opin Struct Biol. 2009;19:31–38. 10.1016/j.sbi.2008.12.003 19157855 PMC2675572

[85] Lynch M, Conery JS. The origins of genome complexity. Science. 2003;302:1401–1404. 10.1126/science.1089370 14631042

[86] Serohijos AWR, Shakhnovich EI. Merging molecular mechanism and evolution: theory and computation at the interface of biophysics and evolutionary population genetics. Curr Opin Struct Biol. 2014;26:84–91. 10.1016/j.sbi.2014.05.005 24952216 PMC4292934

[87] Beran R. Minimum Hellinger distance estimates for parametric models. Ann Statistics. 1977;p. 445–463. 10.1214/aos/1176343842

[88] Kulkarni P, Solomon TL, He Y, Chen Y, Bryan PN, Orban JL. Structural metamorphism and polymorphism in proteins on the brink of thermodynamic stability. Protein Sci. 2018;27:1557–1567. 10.1002/pro.3458 30144197 PMC6194243

[89] He Y, Rozak DA, Sari N, Chen Y, Bryan P, Orban J. Structure, dynamics, and stability variation in bacterial albumin binding modules: implications for species specificity. Biochemistry. 2006;45(33):10102–10109. 10.1021/bi060409m 16906768

[90] Gallagher T, Alexander P, Bryan P, Gilliland GL. Two crystal structures of the B1 immunoglobulin-binding domain of streptococcal protein G and comparison with NMR. Biochemistry. 1994;33(15):4721–4729. 10.1021/bi00181a032 8161530

[91] Eddy SR, et al. A new generation of homology search tools based on probabilistic inference. In: Genome Inform. vol. 23; 2009. p. 205–211. 20180275

[92] Suzek BE, Huang H, McGarvey P, Mazumder R, Wu CH. UniRef: comprehensive and non-redundant UniProt reference clusters. Bioinformatics. 2007;23(10):1282–1288. 10.1093/bioinformatics/btm098 17379688

[93] Bolhuis PG, Chandler D, Dellago C, Geissler PL. Transition path sampling: Throwing ropes over rough mountain passes, in the dark. Ann Rev Phys Chem. 2002;53(1):291–318. 10.1146/annurev.physchem.53.082301.113146 11972010

