# Supplementary material for: Exploring the sequence fitness landscape of a bridge between protein folds
Source: PLoS Comput Biol. 2020 Oct 13;16(10):e1008285. doi: 10.1371/journal.pcbi.1008285 (PMC7553338; doi:10.1371/journal.pcbi.1008285)
Supplement: S1 Text — Procedure for verifying likelihood model via MC simulations (Text A). Wild type and designed amino acid sequences (Table A). Summary of stability and melting temperature of wild-type and designed sequences from previous experiments (Table B). Distribution of energies from Monte Carlo simulations with evolutionary Hamiltonian (Fig. A). Frequencies of amino acids at each position from Monte Carlo simulations with evolutionary Hamiltonian (Fig. B). Correlation between evolutionary Hamiltonian and thermodynamic stability (Fig. C). Dependence of committor ϕA on Ecomb (Fig. D). EA-B of the designed sequences on the GA/GB fold interface (Fig. E). Determining optimal reaction coordinate from sequences with ϕA ≈ 1/2 (Fig. F). Quality of reaction coordinate assessed by comparison of true and calculated ϕA (Fig. G). Transition paths plotted on 2D fitness landscapes (Fig. H). Predictions from DisEMBL predictor (Fig. I). Residues controlling fold switching (Fig. J). (PDF) [file pcbi.1008285.s001.pdf]

S1 Text

Pengfei Tian[1] and Robert B. Best[1]

[1]Laboratory of Chemical Physics, National Institute of Diabetes and Digestive and Kidney Diseases, National Institutes of Health, Bethesda, MD 20892.

Text A

Before proceeding further, we tested whether sequences generated from the likelihood functions recapitulate the properties of the original MSA for each protein. To do this, we ran Metropolis Monte Carlo [1] simulations in sequence space, on either the E_{GA} or E_{GB} energy surface, as detailed in methods. As shown in SI Fig. S1, the energy distribution of the sequences from the MSA of GA or GB is consistent with the sequences generated by the Monte Carlo simulation using energy functions E_{GA} or E_{GB} scaled by a factor of 1.17 or 1.04 respectively. The rescaling of the energy allows us to recover the correct energy distributions by running simulations with each energy function at the same reduced temperature of 1.0. As a more detailed test, we compare the amino acid composition of the sequences from the MSA with those generated by the simulations (SI Fig.S4), finding good agreement

Supporting Tables

Table A: Wild type and designed amino acid sequences.

GA wild-type	MEAVDANSLAQAKEAAIKELKQYGIGDYYIKLINNAKTVEGVESLKNEILKALPTE
$^{63}GA_{MBP}$	NGDKDANSLAEAKEKAIKELKIYGIGEHYIKLIENAKQVEAVESLKDEILKALPRF
$^{64}GA_{MBP}$	NGDKDANSLAEAKEKAIKELKIYGIGEHYIKLIENAKQVAAVESLKDEILKALPRF
$^{68}GA_{MBP}$	NGDKDANSLAEAKEKAIKDLKIYGIGEHYIKLIENAKQVAAVEDLKDEILKALPRF
$^{70}GA_{MBP}$	NGDKDANSLAEAKEKAIKDLKIYGIGEHYIKLIEKAKQVAAVEDLKDEILKALPRF
$^{79}GA_{MBP}$	NGDKGYNGLAEAKEKAIKDLKIYGIGEHYIKLIEKAKQVAAVEDLKDEILKAHDRF
$^{80}GA_{MBP}$	NGDKGYNGLAEAKEKAIKDLKIYGIGEHYIKLIEKAKQVAAVEDLKDIILKAHDRF
GA30	MEAVDANSLAQAKEAAIKELKQYGIGEKYIKLINNAKTVEGVWSLKNEILKALPTE
GA77	TTYKLILNLKQAKEEAIKELVDAGIAEKYIKLIANAKTVEGVWTLKDEILKATVTE
GA77a	TTYKLILNLKQAKEEAIKELVDAAIAEKYIKLIANAKTVEGVWTLKDEILKATVTE
GA77b	TTYKLILNLKQAKEEAIKELVDAGTAEKYIKLIANAKTVEGVWTYKDEILKATVTE
GA77c	TTYKLILNLKQAKEEAIKELVDAGIAEKYFKLIANAKTVEGVWTLKDEILKATVTE
GA77d	TTYKLILNLKQAKEEAIKELVDAGIAEKYIKLIANAKTVEGVWTYKDEILKATVTE
GA77e	TTYKLILNLKQAKEEAIKELVDAGIAEKYIKLIANAKTVEGVWTLKDEIKKATVTE
GA77f	TTYKLILNLKQAKEEAIKELVDAGIAEKYIKLIANAKTVEGVWTLKDEILTATVTE
GA77g	TTYKLILNLKQAKEEAIKELVDAGIAEKYIKLIANAKTVEGVWTLKDEILKFTVTE
GA88	TTYKLILNLKQAKEEAIKELVDAGIAEKYIKLIANAKTVEGVWTLKDEILTFTVTE
GA91	TTYKLILNLKQAKEEAIKELVDAGTAEKYIKLIANAKTVEGVWTLKDEILTFTVTE
GA95	TTYKLILNLKQAKEEAIKELVDAGTAEKYIKLIANAKTVEGVWTLKDEIKTFTVTE
GA98	TTYKLILNLKQAKEEAIKELVDAGTAEKYFKLIANAKTVEGVWTLKDEIKTFTVTE
GB98-T25I	TTYKLILNLKQAKEEAIKELVDAGIAEKYFKLIANAKTVEGVWTYKDEIKTFTVTE
GB98-T25I-L20A	TTYKLILNLKQAKEEAIKEAVDAGIAEKYFKLIANAKTVEGVWTYKDEIKTFTVTE
GB98	TTYKLILNLKQAKEEAIKELVDAGTAEKYFKLIANAKTVEGVWTYKDEIKTFTVTE
GB98a	TTYKLILNLKQAKEEAIKELVDAGTAEKYFKLYANAKTVEGVWTYKDEIKTFTVTE
GB95	TTYKLILNLKQAKEEAIKEAVDAGTAEKYFKLIANAKTVEGVWTYKDEIKTFTVTE
GB91	TTYKLILNLKQAKEEAIKEAVDAGTAEKYFKLYANAKTVEGVWTYKDEIKTFTVTE
GB88	TTYKLILNLKQAKEEAITEAVDAGTAEKYFKLYANAKTVEGVWTYKDEIKTFTVTE
GB77g	TTYKLILNGKQLKEEAITEAVDAATAEKYFKLYANAKTVEGVWTYKDEIKTFTVTE
GB77f	TTYKLILNGKQLKEEAITEAVDAATAEKYFKLIANAKTVEGVWTYKDETKTFTVTE
GB77e	TTYKLILNGKQLKEEAITEAVDAAIAEKYFKLYANAKTVEGVWTYKDETKTFTVTE
GB77d	TTYKLILNGKQLKEEAITEAVDAGTAEKYFKLYANAKTVEGVWTYKDETKTFTVTE
GB77c	TTYKLILNGKQLKEEAITELVDAATAEKYFKLYANAKTVEGVWTYKDETKTFTVTE
GB77b	TTYKLILNGKQLKEEAIKEAVDAATAEKYFKLYANAKTVEGVWTYKDETKTFTVTE
GB77a	TTYKLILNLKQAKEEAITEAVDAATAEKYFKLYANAKTVEGVWTYKDETKTFTVTE
GB77	TTYKLILNGKQLKEEAITEAVDAATAEKYFKLYANAKTVEGVWTYKDETKTFTVTE
GB30	MTYKLILNGKTLKGETTTEAVDAATAEKYFKLYANDKTVEGEWTYDDATKTFTVTE
GB wild type	MTYKLILNGKTLKGETTTEAVDAATAEKVFKQYANDNGVDGEWTYDDATKTFTVTE

Table B: Summary of stability and melting temperature of wild-type and designed sequences from previous experiments[2, 3, 4, 5].

Protein	ΔG 25°C	ΔG 20°C	T_m/°C	Protein	ΔG 25°C	ΔG 20°C	T_m/°C
GAwt[2]	6		86.0	GBwt[2]	7		87.5
GA30[2]	6		86.0	GB30[2]	4.5		65.0
GA77[2]	5	5	77.5	GB77[2]	4.0	5	62.4
GA88[2]	4		69.4	GB88[2]	2		57.5
GA91[3]		4	61.5	GB91[3]		3.1	49.3
GA95[3]		~ 3	50.0	GB95[3]		~ 3	48.7
GA98[3]		1.5	37.0	GB98[3]		2.0	35.0
GA77d[3]		3.5	60.2	GB98a[3]			37.0
GA77g[3]		4.7	75.3	GB98-T25I[4]			36.0
GA77a[3]			65.8	GB77a[3]			63.8
GA77b[3]			67.3	GB77b[3]			55.8
GA77c[3]			62.5	GB77c[3]			49.9
GA77e[3]			65.5	GB77d[3]			58.3
GA77f[3]			71.6	GB77e[3]			58.0
$^{63}GA_{MBP}$ [5]			80.0	GB77f[3]			61.9
$^{64}GA_{MBP}$ [5]			76.0	GB98-T25I-L20A [4]			46.0
$^{68}GA_{MBP}$ [5]			62.0				
$^{70}GA_{MBP}$ [5]			63.0				
$^{79}GA_{MBP}$ [5]			55.0				
$^{80}GA_{MBP}$ [5]			53.0				

Supporting Figures

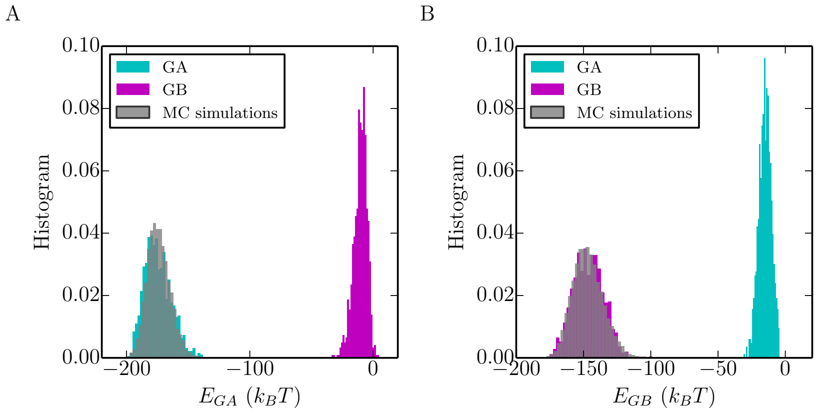

Fig. A: Distribution of E_{GA} (a) and E_{GB} (b) of homologous sequences from GA (cyan) and GB (purple) alignments. The distribution of E_{GA} and E_{GB} of the sequences sampled during Monte Carlo simulations are shown in grey. In the simulation, the energy functions of E_{GA} and E_{GB} have been scaled by a factor of 1.17 and 1.04 respectively, in order that the sampled distributions of energies match those from the original sequences.

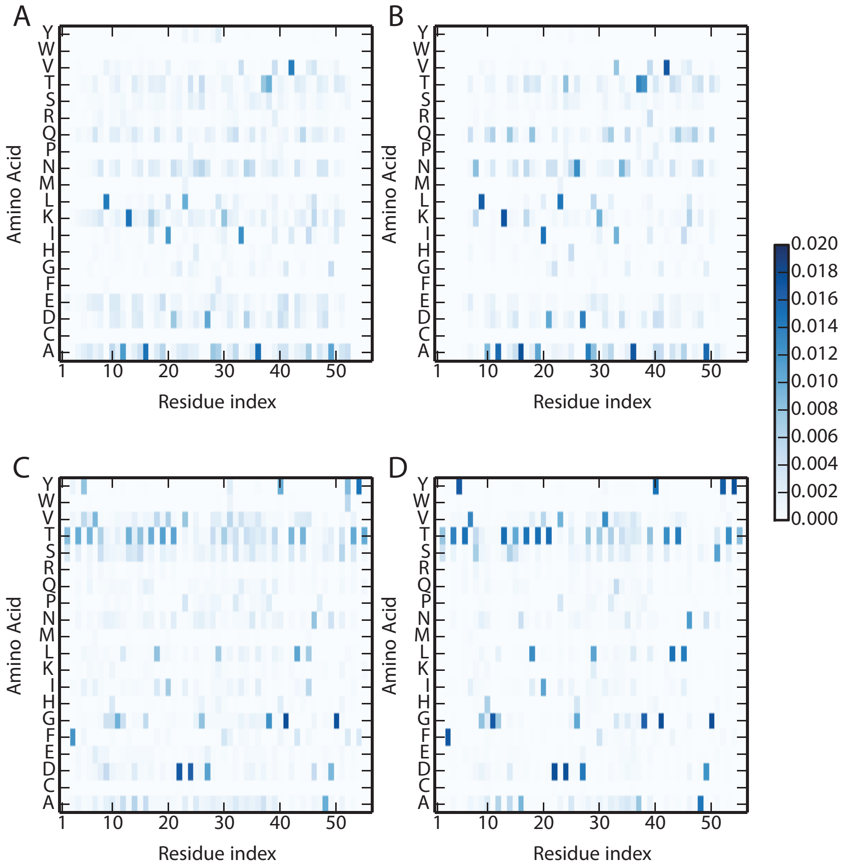

Fig. B: The single-site amino acid occupancies in the multiple sequence alignment of GA (A) are reproduced by the sequences generated by Monte Carlo simulations (B). Likewise, the single-site amino acid occupancies in the multiple sequence alignment of GB (C) are reproduced by sequences generated by MC simulations (D).

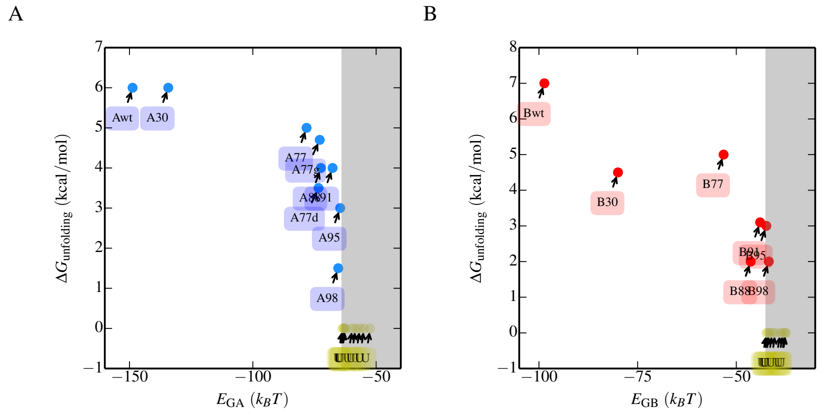

Fig. C: Evolutionary Hamiltonian and thermodynamic stability. The relation between stability $\Delta G_{\rm unfolding}$ and evolutionary Hamiltonian of GA and GB is shown in (A) and (B) respectively. $\Delta G_{\rm unfolding}$ are measured in the previous experiments [2, 3, 4].

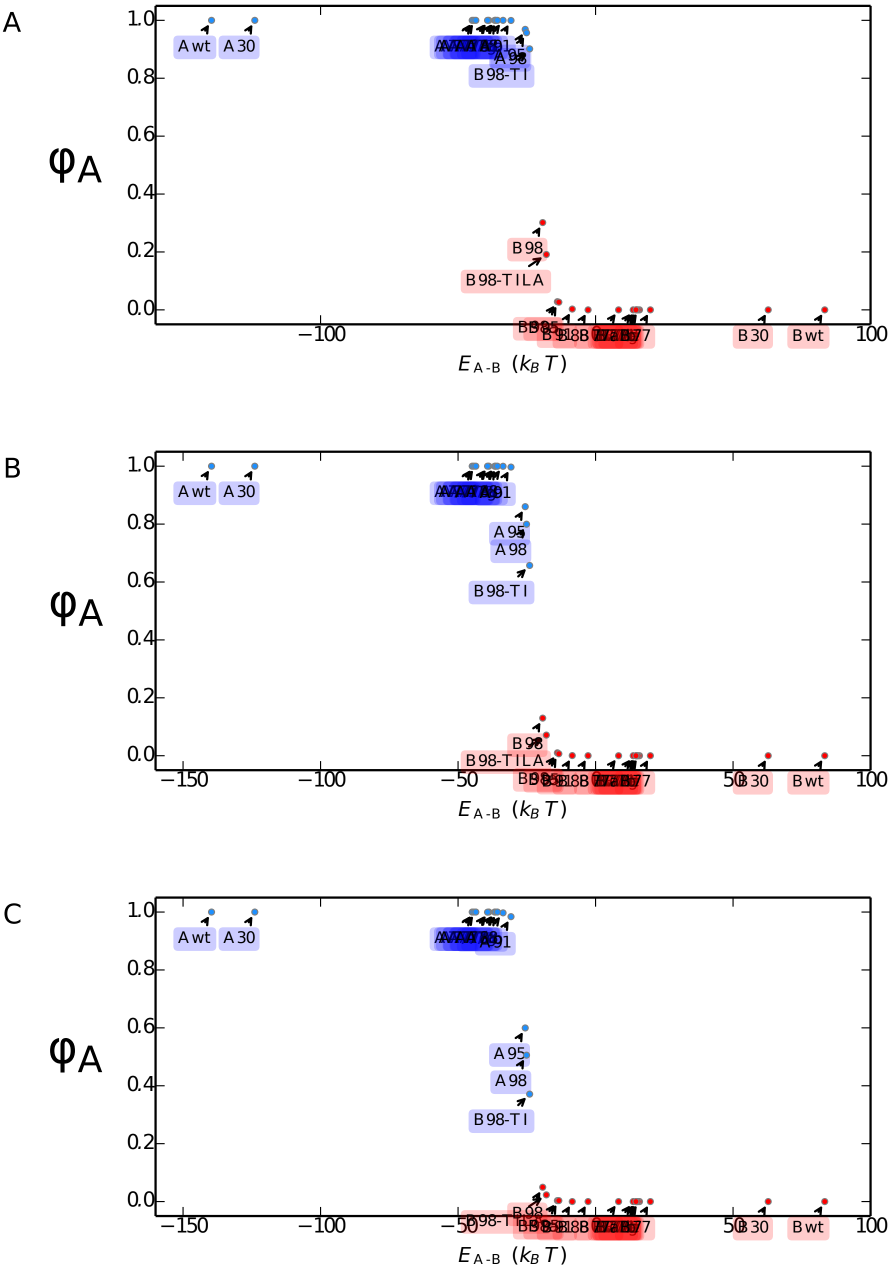

Fig. D: ϕ_A of mutations estimated by the first passage simulations. ϵ values of 21.0, 23.0, 25.0 are used in the figure A), B) and C) respectively.

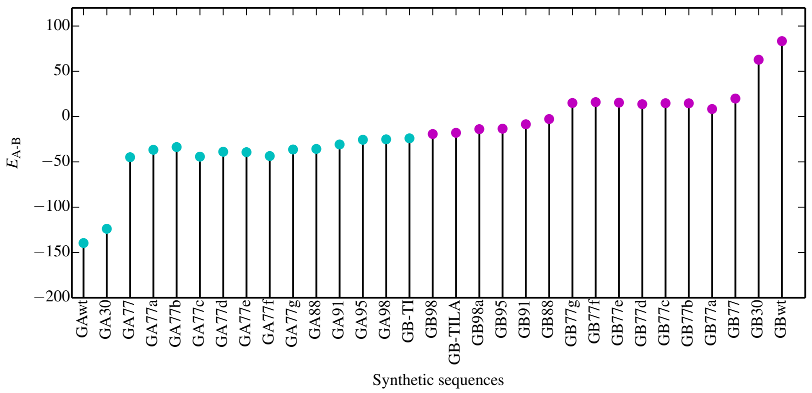

Fig. E: $E_{\text{A-B}}$ of the designed sequences on the GA/GB fold interface.

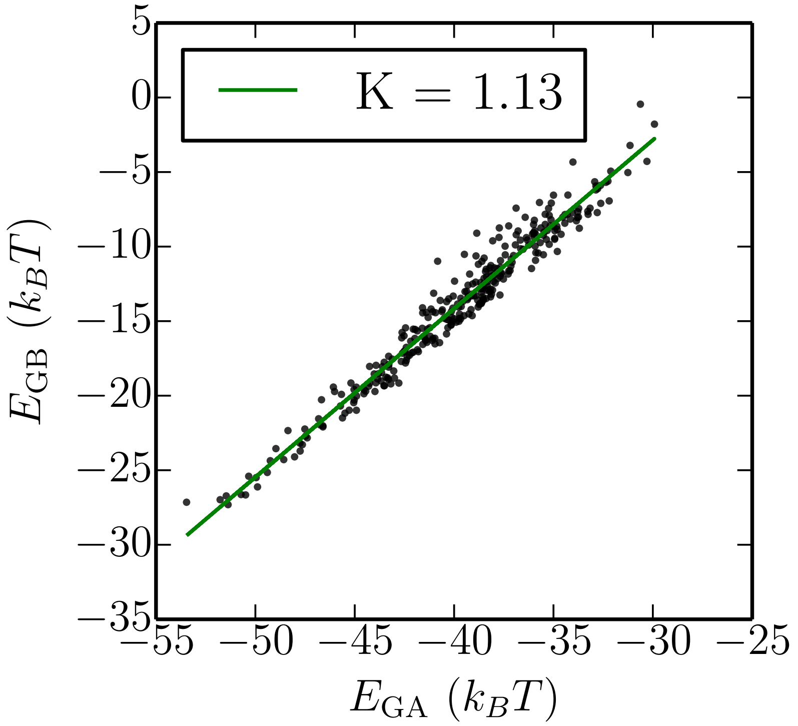

Fig. F: The designed sequences with $P_{\text{fold}} \in [0.48, 0.52]$ are projected on the two coordinates E_{GA} and E_{GB}. The least square fitting gives a straight line of slope K = 1.13.

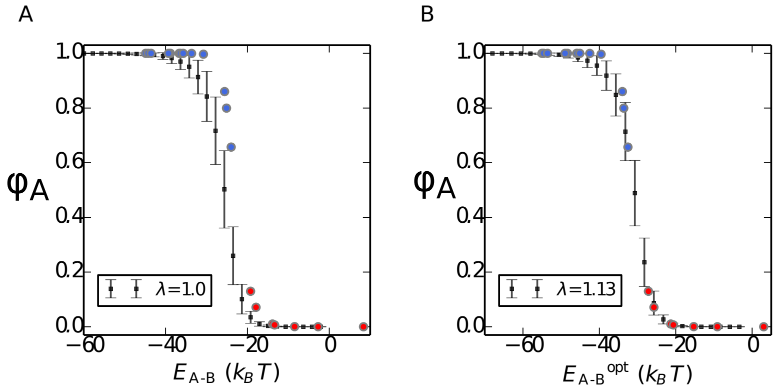

Fig. G: The mean and standard deviation of ϕ_A is calculated along the reaction acoordinate $E_{\text{A-B}}$ with $\lambda = 1.0$ and the optimized one $E^{\text{opt}}_{\text{A-B}}$ with λ = 1.15.

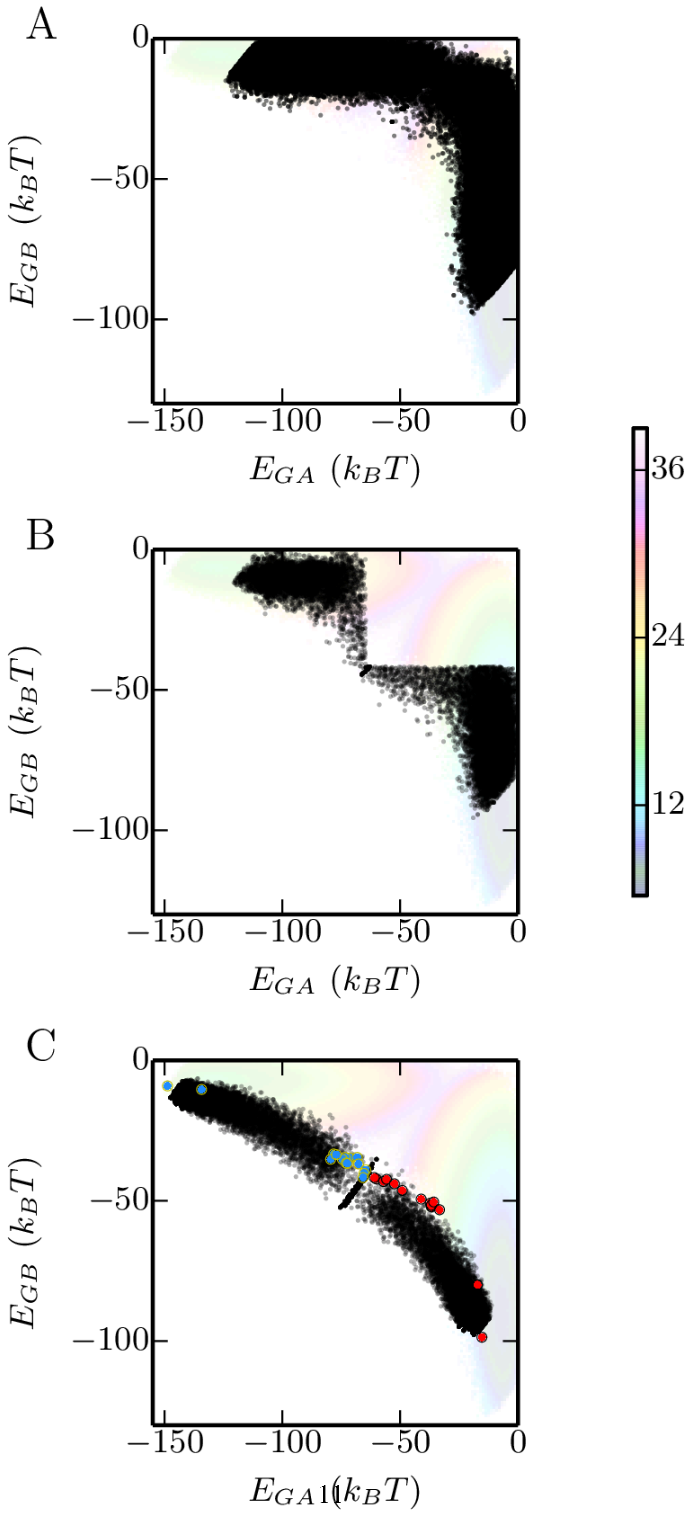

Fig. H: The transition path sequences are plotted in black dots for the case of natural mutation (A), natural mutations with stability constraints (B), and binary mutations (C). Red and blue dots are experimental mutations with GA and GB folds respectively.

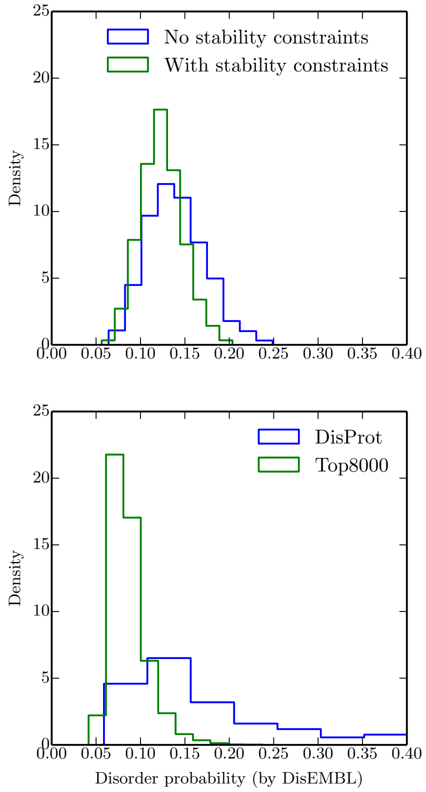

Fig. I: Predictions from DisEMBL predictor [6]. Top: distribution of mean DisEMBL scores for GA/GB switch sequences with or without a stability constraint. Bottom: distribution of mean DisEMBL scores for the Top8000 structure database [7] and the DisProt sequence database of disordered sequence [8].

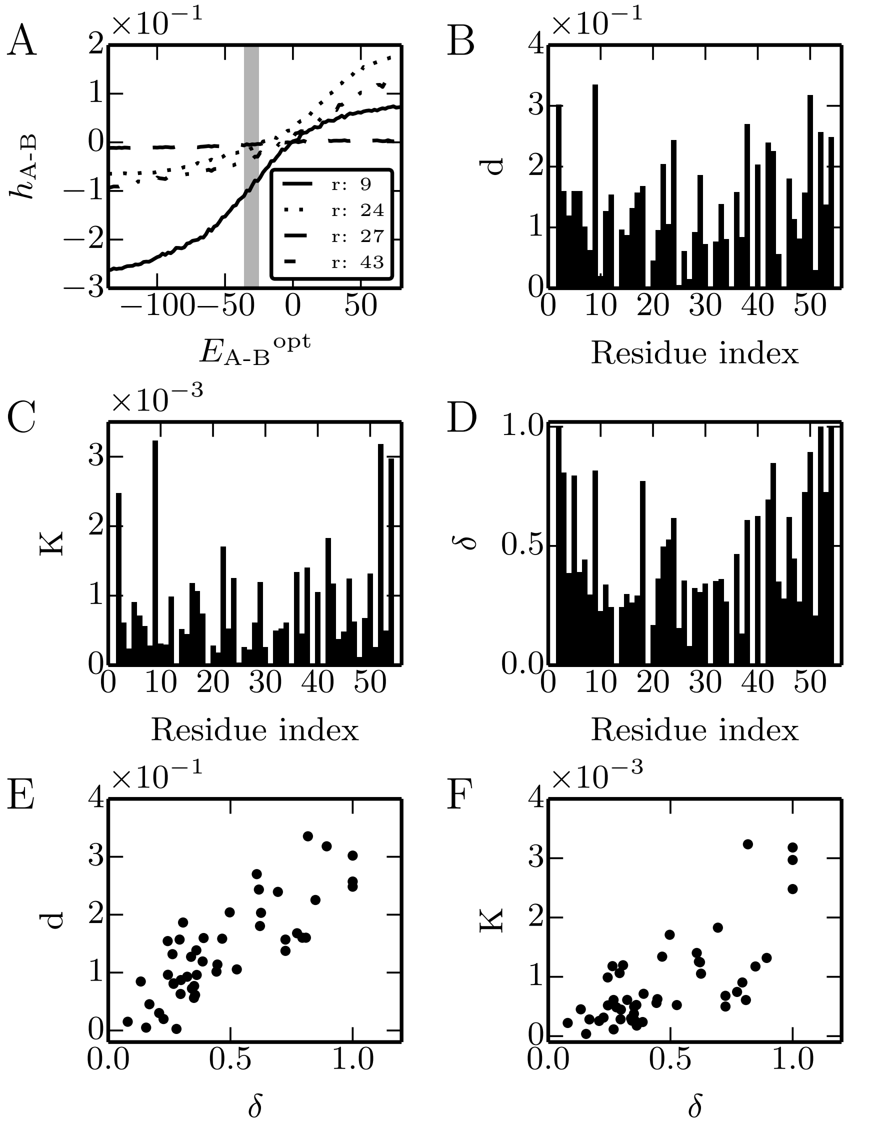

Fig. J: Natural mutations. (A) examples of Hb-Ha for residues 20, 25, 45. (B) Total change (d) of Hb-Ha from GA to GB. (C) Slope K at transition. (D) The difference (δ) in residue propensity between GA and GB homologs. (E) Correlation of d with δ. (F) Correlation of slope with propensity.

References

1. Metropolis N, Metropolis AW, Rosenbluth MN, Teller AH, Teller E. Equation of state calculation by fast computing machines. J Chem Phys. 1953;21(6):1087.

2. Alexander PA, He Y, Chen Y, Orban J, Bryan PN. The design and characterization of two proteins with 88% sequence identity but different structure and function. Proc Natl Acad Sci U S A. 2007;104(29):11963–11968.

3. Alexander PA, He Y, Chen Y, Orban J, Bryan PN. A minimal sequence code for switching protein structure and function. Proc Natl Acad Sci U S A. 2009;106(50):21149–21154.

4. He Y, Chen Y, Alexander PA, Bryan PN, Orban J. Mutational tipping points for switching protein folds and functions. Structure. 2012;20(2):283–291.

5. Porter LL, He Y, Chen Y, Orban J, Bryan PN. Subdomain interactions foster the design of two protein pairs with 80% sequence identity but different folds. Biophys J. 2015;108(1):154–162.

6. Linding R, Jensen LJ, Diella F, Bork P, Gibson TJ, Russel RB. Protein disorder prediction: implications for structural proteomics. Structure. 2003;11:1453–1459.

7. Chen VB, Arendall WB, Headd JJ, Keedy DA, Immormino RM, Kapral GJ, et al. MolProbity: all-atom structure validation for macromolecular crystallography. Acta Crystallographica Section D: Biological Crystallography. 2010;66(1):12–21.

8. Sickmeier M, Hamilton JA, LeGall T, Vacic V, Cortese MS, Tantos A, et al. DisProt: the database of disordered proteins. Nucleic Acids Res. 2007;35(suppl 1):D786–D793.
